# Signaling pathways in macrophages: molecular mechanisms and therapeutic targets

**DOI:** 10.1002/mco2.349

**Published:** 2023-09-11

**Authors:** Ming Li, Mengjie Wang, Yuanjia Wen, Hongfei Zhang, Guang‐Nian Zhao, Qinglei Gao

**Affiliations:** ^1^ Department of Gynecological Oncology Tongji Hospital Tongji Medical College Huazhong University of Science and Technology Wuhan China; ^2^ National Clinical Research Center for Obstetrics and Gynecology Cancer Biology Research Center (Key Laboratory of the Ministry of Education) Tongji Hospital Tongji Medical College Huazhong University of Science and Technology Wuhan China

**Keywords:** diseases, macrophage, signaling pathway, therapeutic targets

## Abstract

Macrophages play diverse roles in development, homeostasis, and immunity. Accordingly, the dysfunction of macrophages is involved in the occurrence and progression of various diseases, such as coronavirus disease 2019 and atherosclerosis. The protective or pathogenic effect that macrophages exert in different conditions largely depends on their functional plasticity, which is regulated via signal transduction such as Janus kinase–signal transducer and activator of transcription, Wnt and Notch pathways, stimulated by environmental cues. Over the past few decades, the molecular mechanisms of signaling pathways in macrophages have been gradually elucidated, providing more alternative therapeutic targets for diseases treatment. Here, we provide an overview of the basic physiology of macrophages and expound the regulatory pathways within them. We also address the crucial role macrophages play in the pathogenesis of diseases, including autoimmune, neurodegenerative, metabolic, infectious diseases, and cancer, with a focus on advances in macrophage‐targeted strategies exploring modulation of components and regulators of signaling pathways. Last, we discuss the challenges and possible solutions of macrophage‐targeted therapy in clinical applications. We hope that this comprehensive review will provide directions for further research on therapeutic strategies targeting macrophage signaling pathways, which are promising to improve the efficacy of disease treatment.

## Graphical abstract

In this review, the authors introduce the research advances related to macrophage signaling pathways by first providing an overview of the basic physiology of macrophages and expound on the regulatory pathways within them. Then, the pathogenic effect of macrophages and the therapeutic strategies targeting macrophage signaling pathways are addressed in various diseases.

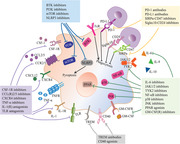

## 1 INTRODUCTION

Tissue homeostasis refers to the state of dynamic balance of tissue structure and function, the destruction of which leads to the occurrence of diseases.^1^, ^2^ The maintenance of tissue homeostasis relies on a series of sensors, signals, and effectors.^2^ Deviation of the regulatory variable from the normal range can activate sensors, which leads to the generation of signals acting on effectors, thereby correcting the deviation of the regulatory variable.^2^ Macrophages are professional sensors due to their wide distribution in almost every organ tissue and their marvelous adaptability to diverse stimuli.^3^, ^4^

Plasticity is one of the hallmarks of macrophages, which refers to the ability to change their phenotypes, functions, and physiological characteristics in response to various external challenges.^3^, ^5^ This is one of the underlying reasons why macrophages can exert multiple key effects in organisms. Transcriptional regulation of gene expression underlies macrophage plasticity, and the bridge between stimulation and transcription is a complex network of signaling pathways involving activation and inhibition of signaling cascade responses.^5^, ^6^ The perception and integration of information by macrophages result in extensive activation and significant crosstalk of signaling pathways, resulting in delicate and subtle responses to environmental changes.

Unfortunately, macrophage plasticity is not only involved in organism protection but also has pathogenic effects.^7^ In some conditions, various stimuli lead to abnormal macrophage activation or dysfunction, resulting in a causal association between macrophages and disease states.^8^ The identification of molecules and signaling pathways involved in macrophage plasticity and activation provide the basis for macrophage‐centered therapeutic strategies.

In this review, we first outline the latest understanding of the basic physiology of macrophages, including their origins, replenishment, polarization, and physiological functions. Then, we focus on important signaling pathways regulating the functions of macrophages in a receptor‐centered way. On this basis, we discuss the current therapeutic approaches in clinical use or under clinical trials that target key components of these signaling pathways, including ligands, receptors and effectors, in different diseases. Finally, we discuss the challenges and future research directions of macrophage‐targeted therapy.

## 2 BASIC PHYSIOLOGY AND ROLE OF MACROPHAGES

Macrophages, originally identified because of their phagocytic nature, are a heterogeneous group of immune cells that are widely distributed throughout the organism and play important roles in development and homeostasis maintenance. Furthermore, it is expected that the origins and polarization of macrophages will determine their phenotypes and functions in specific microenvironments. In this section, we will provide an overview of the basic biology of macrophages, including their origins, replenishment, polarization, and physiological functions.

### 2.1 Origins of macrophages

Although macrophages were initially thought to originate primarily from hematopoietic stem cells (HSCs) and circulating monocytes,^9^ accumulating evidence has clarified an embryo‐derived macrophage lineage with macrophage precursors derived from erythro‐myeloid progenitors (EMPs) in yolk sacs and fetal liver.^9^, ^10^ The EMP‐derived macrophage precursors can settle throughout the embryo and form subpopulations of tissue‐specific macrophages during organogenesis. And these tissue‐specific macrophages can establish stable spatial and functional relationships with specialized tissue cells and are termed “tissue‐resident macrophages (TRMs).”^11^ The EMPs then disappear during fetal life, but EMP‐derived TRMs persist and self‐renew in adults, such as microglia, Kupffer cells, and Langerhans cells.^12^ In addition, a small proportion of TRMs are gradually replaced or replenished by HSCs‐derived macrophages. For example, alveolar macrophages and osteoclasts are derived from EMPs and HSCs in adults,^11^ while the mucosal macrophages in the gut lamina propria are derived from HSCs and circulating monocytes in adults.^13^ Furthermore, macrophages functioning in inflammatory or other pathological states are predominantly derived from circulating monocytes.^5^, ^14^, ^15^

### 2.2 Replenishment of macrophages

Macrophages must maintain or increase their population to better perform functions, and two strategies are at stake.^14^, ^16^ One is the recruitment of monocytes, and the other is to increase the proliferation of TRMs by enhancing their self‐renewal capacity.^14^, ^17^

#### 2.2.1 Recruitment of macrophages

Under tissue stress conditions, including inflammation and cancer, circulating monocytes can be recruited to specific sites and differentiate into macrophages with specific functions. This process relies on the participation of chemokines or cytokines.^18^ C‐C motif ligand (CCL) 2 and CCL5 specifically attract and activate monocytes and are key players in the regulation of monocyte/macrophage migration and infiltration.^19^, ^20^ Colony‐stimulating factor‐1 (CSF‐1) and vascular endothelial growth factor (VEGF) have potent chemotactic effects on monocytes and macrophages via CSF‐1 receptor (CSF‐1R) and VEGF‐R1, respectively.^21^, ^22^ Endothelial monocyte‐activating polypeptide II was found to promote chemotaxis of monocytes and macrophages, with endothelins (ET)‐1 having a chemotactic effect on human monocytes and ET‐2 on macrophages.^19^

#### 2.2.2 In situ proliferation of macrophages

Recent evidence points to the self‐renewal and numerical maintenance of macrophages in adult tissues under homeostatic conditions by in situ proliferation of TRMs rather than an influx of hematopoietic progenitors.^23^, ^24^ It has been suggested that local proliferation of TRMs is more involved in tissue macrophage regeneration after mild injury, whereas monocyte‐derived macrophages are more engaged in severe conditions.^3^, ^25^ Notably, TRMs gradually lose their self‐renewal ability with aging.^26^ However, the mechanisms underlying the local proliferation and self‐renewal ability of TRMs are still being explored.

### 2.3 Polarization and functional phenotypes of macrophages

Polarization of macrophages is a process of altering phenotypes and function in response to microenvironmental stimuli,^27^ which is essential for macrophages to function appropriately in different environments.^28^, ^29^

M1 and M2 macrophages represent the two main subpopulations of macrophages.^29^ M1 macrophages, also called classically activated macrophages, express molecular markers such as CD80, CD86, CD68, major histocompatibility complex class II (MHC‐II), and Toll‐like receptor (TLR) 4,^29^ which are activated by lipopolysaccharide (LPS), interferon‐γ (IFN‐γ), or tumor necrosis factor α (TNF‐α). M1 macrophages secrete a variety of proinflammatory cytokines^30,^ such as interleukin (IL)‐1, IL‐6, TNF‐α, nitric oxide (NO), reactive oxygen species (ROS), and downregulate the expression of *IL‐12*, *IL‐23*, and *IL‐10*.^31^ M1 macrophages mainly promote Th1 immune responses, possess potent antimicrobial and antitumor effects, and mediate ROS‐induced tissue damage that impairs tissue regeneration and wound healing.^31^

To avoid tissue damage and maintain homeostasis, anti‐inflammatory M2 macrophage‐driven regulatory mechanisms suppress chronic inflammatory responses.^30^ M2 macrophages, also known as alternatively activated macrophages, express surface markers, including CD206, CD163, CD209, FIZZ1, Ym1/2 and galactose receptor.^29^, ^32^ M2 macrophages are mainly activated by CSF‐1, IL‐4, IL‐13, and transforming growth factor‐β (TGF‐β) and can secrete IL‐10, arginase 1 (Arg 1) and TGF‐β, thus playing important roles in inflammation inhibition, tissue remodeling, wound healing, angiogenesis, the T helper type 2 (Th2) immune response, and immune regulation.^31^, ^32^

As the study progressed, it was established that macrophage polarization is on a continuum, and in addition to M1 and M2 macrophages, there exist other phenotypes of macrophages, such as CD169+ and T cell receptor positive (TCR+) macrophages.^33^ CD169+ macrophages are a unique subpopulation of macrophages predominantly located in lymphoid organs such as lymph nodes and spleen. They express molecular markers such as CD169, CD206, and vascular cell adhesion molecule‐1 (VCAM‐1). CD169+ macrophages have the ability to release CCL22 and participate in erythropoiesis, immune tolerance, antigen presentation, and immune regulation.^33^, ^34^, ^35^ TCR+ macrophages express TCRαβ or γδ and CD3. They also express molecules necessary for TCR signaling on lymphocytes, such as ZAP70 and Fyn, but the concentrations of these molecules are different when macrophages are stimulated by IL‐4 or IFN‐γ. TCR+ macrophages can release CCL2 and have a high phagocytosis capacity, playing a role in inflammation and infectious diseases.^33^

In conclusion, macrophages are heterogeneous populations composed of multiple subpopulations with different phenotypes and functions, each with their own characteristics and functions. In vivo, macrophages can flexibly adopt specific functional phenotypes in response to subtle and continuous variations in the tissue microenvironment and then play an important role in development and homeostasis.^14^

### 2.4 The role of macrophages in development and homeostasis

Macrophages play essential roles in all stages of development.^36^ For instance, during embryogenesis, macrophages derived from yolk sac progenitors contribute to tissue structural reconstruction through the elimination of apoptotic cells.^4^, ^36^ The development and maintenance of the central nervous system (CNS) depend on the precise regulation of microglia.^37^, ^38^ In addition, macrophages also serve important functions in angiogenesis,^39^, ^40^ lymph angiogenesis,^41^ mammary duct branching and pancreas islet formation,^42^, ^43^ adipogenesis, myocyte development and growth, and erythropoiesis.^3^, ^8^

Macrophages utilize their powerful biological functions to play diverse roles in tissue homeostasis maintenance as well.^4^, ^7^, ^8^ Macrophages block the occurrence of tissue inflammation and injury by phagocytic clearance of debris, damaged cells, dead cells and apoptotic cells.^29^, ^44^ When tissue damage occurs, TRMs can regulate extracellular matrix formation to effectively repair the tissue and thus maintain the stable state of the tissue.^45^, ^46^, ^47^ Macrophages also serve an essential immune function, as they can recognize invasive microbes or tumor cells, exert direct killing effects,^48^ and transmit signals to other immune cells to respond.^49^, ^50^ Moreover, macrophages are involved in the tissue metabolism of calcium,^51^ iron,^52^ bilirubin, amino acids, and lipids,^53^ regulating the stable level of these metabolites required in organs to ensure their normal function.^4^

## 3 SIGNALING PATHWAYS IN MACROPHAGES

The diversity of macrophage functions depends on their plasticity in response to environmental stimuli. In response to diverse microenvironmental signals or under distinct pathophysiological conditions, macrophages can obtain different functional phenotypes through polarization. This process is regulated by various ligand–receptor recognitions through receptor‐mediated signaling transduction and downstream signaling pathways. The integration of different stimulus signals and the crosstalk and balance among signaling pathways ultimately determine the phenotype and function of macrophages. And these ligands, receptors, and signaling pathways have been shown to serve as targets for disease treatment. Thus, we then introduce these important signaling pathways mainly in a receptor‐centered way.

### 3.1 Pattern recognition receptor (PRR)‐associated signaling pathways

#### 3.1.1 TLRs

**FIGURE 1:**
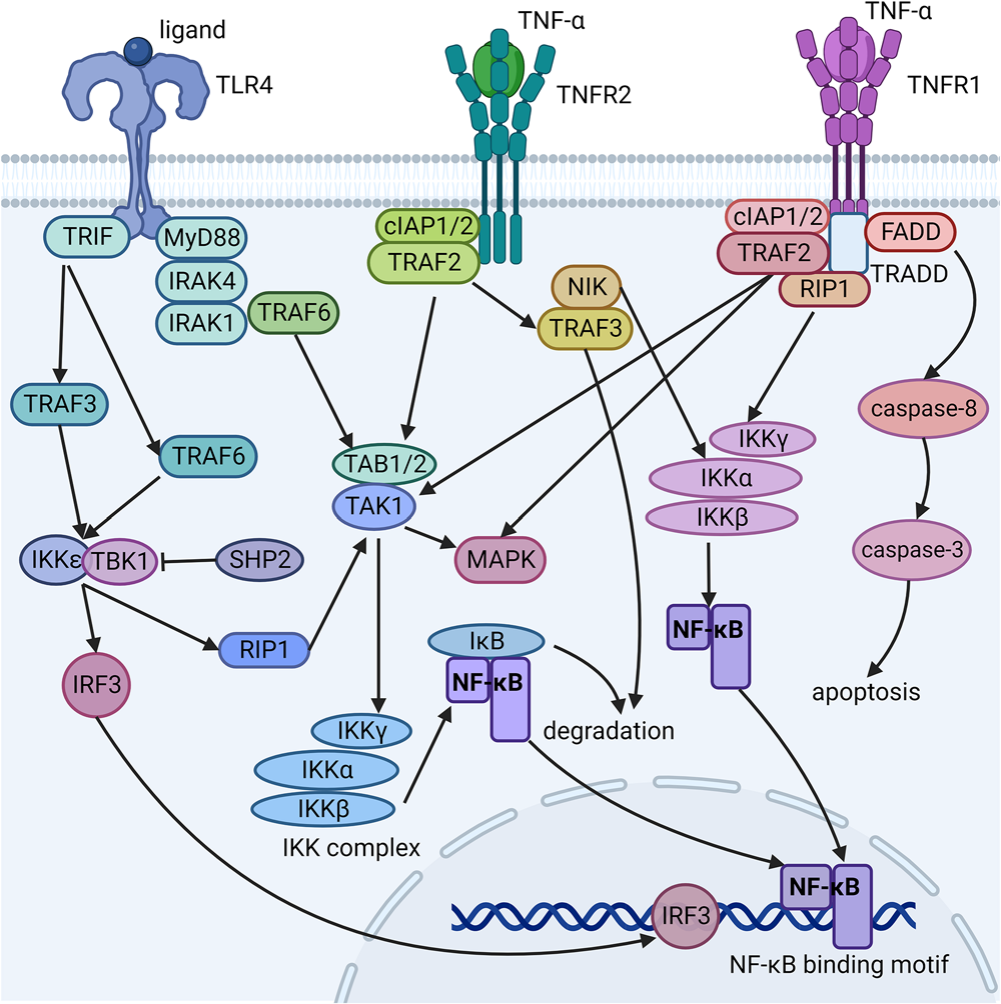
Activation of the NF‐κB signaling pathway in macrophages. Activation of NF‐κB in macrophages is mainly associated with pro‐inflammatory cytokines such as TNF‐α, or recognition of microbial products by TLRs. These ligand–receptor interactions converge on IKK activation through diverse upstream signaling pathways. The IKK complex, consisting of IKKα, IKKβ, and IKKγ, can degrade IκB and thereby activate NF‐κB. Activated NF‐κB mediates the expression of a series of pro‐inflammatory cytokine and chemokine genes. In addition, TLR4 can mediate type I interferon expression by activating IRF3 through TRIF, and TNF‐α mediates caspase‐3‐dependent apoptosis by activating FADD through TNFR1. Abbreviations: TLR, Toll‐like receptor; MyD88, myeloid differentiation primary‐response protein 88; TRIF, TIR domain‐containing adaptor protein inducing IFN‐β; IRAK, IL‐1R‐associated kinase; TRAF, tumor‐necrosis factor (TNF)‐receptor‐associated factor; TAK, transforming‐growth‐factor‐β‐activated kinase; TAB, TAK1‐binding protein 1; TBK, TRAF‐family‐member‐associated NF‐κB activator‐binding kinase 1; IKK, inhibitor of nuclear factor‐κB (IκB)‐kinase; SHP2, Src homology 2 (SH2) domain‐containg protein tyrosine phosphatase; RIP1, receptor‐interacting protein 1; IRF3, IFN‐regulatory factor 3; cIAP1/2, cellular inhibitors of apoptosis1/2; NIK, NF‐κB‐inducing kinase; TNF‐α, tumor necrosis factor‐α; TNFR, TNF receptor; TRADD, TNFR1‐associated death domain protein; FADD, Fas‐associated death domain; MAPK, mitogen‐activated protein kinases; IκB, inhibitor of nuclear factor‐κB; NF‐κB, nuclear factor kappa‐B. (Created with BioRender.com).

TLRs are important PRRs expressed in macrophages^54^ that regulate the survival, activation, and function of macrophages by two distinct signaling pathways, the myeloid differentiation primary‐response protein 88 (MyD88)‐dependent pathway and the TLR/IL‐1R (TIR) domain‐containing adaptor protein inducing IFN‐β (TRIF)‐dependent pathway (Figure 1).^54^, ^55^

Upon binding to ligands, some TLRs, such as TLR2, TLR4, and TLR6, recruit MyD88, which binds to the IL‐1R‐associated kinase (IRAK) 4 complex, leading to the autophosphorylation of IRAK1.^54^ Phosphorylated IRAK1 binds to TNF‐receptor‐associated factor (TRAF) 6, and then both detach from the receptor complex to form a new complex with TGF‐β‐activated kinase (TAK) 1, TAK1‐binding protein (TAB) 1 and TAB2 on the plasma membrane. Then, IRAK1 is degraded, while the rest of the complex is translocated to the cytoplasm, where TRAF6 interacts with ubiquitin‐conjugating enzyme 13 (UBC13) and UBC E2 variant 1 to activate TAK1. TAK1 activates mitogen‐activated protein kinase (MAPK) and then activator protein‐1 (AP‐1) to induce the transcription of *IL‐1*, *IL‐6*, *TNF‐α*, and other inflammatory factor genes.^56^ TAK1 also phosphorylates the nuclear factor‐κB (IκB)‐kinase (IKK) complex to promote the nuclear translocation of nuclear factor kappa‐B (NF‐κB) and the expression of target genes.^56^, ^57^

TLR3 and TLR4 can also activate the TRIF‐dependent pathway.^54^ TLR3/4 recruits TRIF, which binds to TRAF3/6. Then, TRAF3 recruits TBK1 and IKKε, which leads to the phosphorylation and nuclear translocation of IFN regulatory factor (IRF) 3, inducing the expression of type I IFN.^56^ TRIF can also activate MAPK and NF‐κB signaling via UBC13/TAK1 by binding to TRAF6 and the serine/threonine kinase receptor‐interacting protein 1 (RIP1).^57^, ^58^

#### 3.1.2 Nucleotide‐binding oligomerization domain‐like receptors (NLRs)

The NLR family includes 22 members of three subfamilies, NLR family C‐terminal caspase activation and recruitment domain (CARD) containing protein (NLRC), NLR thermal protein domain associated protein (NLRP), and ICE‐protease activating factor (IPAF), among which NLRP3^59^ and NLRC3^60^ play regulatory roles in macrophage polarization and the inflammatory response.

Upon stimulation by multiple pathogen‐associated molecular patterns or damage‐associated molecular patterns, NLRP3 oligomerizes and recruits apoptosis‐associated speck‐like protein containing a C‐terCARD (ASC) to form ASC speck through its PYD domain.^61^ Then, ASC speck and pro‐caspase‐1 form the NLRP3 inflammasome via CARD–CARD interactions, promoting the maturation of caspase‐1 through pro‐caspase‐1 self‐cleaving.^62^ Caspase‐1 cleaves pro‐IL‐1 and pro‐IL‐18 to form activated IL‐1β and IL‐18 secreted extracellularly to mediate the inflammatory response.^62^ Caspase‐1 also releases the activity of GSDMD^Nterm^ by removing the carboxyl terminus of gasdermin D (GSDMD).^63^ Then, GSDMD^Nterm^ binds to the cell membrane to form membrane pores, leading to cell death and the release of large amounts of proinflammatory factors.^64^ This process, known as pyroptosis, is an inflammatory programmed cell death that plays a role in pathogen clearance by macrophages.^64^ In addition, NLRP3 promotes NO production by inducible NO synthase (iNOS).^65^

In contrast to NLRP3, NLRC3 inhibits macrophage activation and prevents inflammatory factor production by negatively regulating multiple inflammatory pathways.^60^ For example, NLRC3 can inhibit the activation of TRAF6 and IRAK1 and promote their degradation, thus inhibiting the activation of NF‐κB signaling.^66^ In addition, NLRC3 can disrupt the formation of inflammasomes by blocking the ASC and pro‐caspase‐1 interaction through the CARD domain.^67^

#### 3.1.3 Cyclic GMP‐AMP synthase (cGAS)‐stimulator of IFN genes (STING)

The cGAS‐STING signaling pathway regulates macrophage polarization and the production of various cytokines, which play a role in pathogen clearance, the inflammatory response, and antitumor immunity.^68^, ^69^

As an important PRR, cGAS is a direct sensor of DNA in host cells. cGAS can recognize and bind to cytoplasmic dsDNA to undergo conformational changes and activation and then catalyze the synthesis of cyclic GMP‐AMP (cGAMP) from intracellular adenosine triphosphate (ATP) and guanosine triphosphate (GTP). cGAMP acts as a second messenger to bind to STING, a linker protein anchored in the endoplasmic reticulum (ER), causing conformational changes and activation.^68^ In addition, cyclic dinucleotides produced by bacteria^68^, ^70^ can directly activate STING. Subsequently, activated STING translocates from the ER to the Golgi, where it undergoes palmitoylation and activates TBK1 and IFN‐regulatory factor 3 to induce the strong expression of type I IFNs.^71^, ^72^ STING can also activate the NF‐κB signaling pathway through molecules such as TRAF6,^73^ IKK,^74^ TKB1,^75^ and signal transducer and activator of transcription 6 (STAT6).^76^

### 3.2 TNFR‐related signaling pathways

TNF‐α plays an important role in regulating macrophage proliferation, apoptosis, and inflammation by activating signaling pathways, including NF‐κB and MAPK, through binding to its receptor TNFR1/2 (Figure 1).^77^ TNFR1 recruits TNFR1‐associated death domain protein (TRADD) through its death domain, which is isolated by the silence of death domain in the absence of TNF‐α.^78^ TRADD attracts Fas‐associated death domain (FADD), TRAF2, and RIP1.^78^, ^79^ FADD mediates apoptosis by sequentially activating caspase‐8 and caspase‐3 through FADD‐like IL‐1β‐converting enzyme. TRAF2 binds ubiquitin ligases cellular inhibitors of apoptosis (cIAPs) 1/2, contributing to polyubiquitin chain assembly of diverse signaling molecules, including NF‐κB‐inducing kinase (NIK), RIP1, TRAF2, and cIAP itself.^80^, ^81^ TRAF2 can also activate IKK, thereby activating NF‐κB and promoting the expression of proinflammatory cytokines and other target genes.^77^ In addition, TRAF2 also activates MAPK kinase kinase 1 (MEKK1), a MAP3K that activates c‐Jun kinases (JNKs) and p38 MAPK through MAP2K4/7, regulating macrophage inflammation and survival.^79^, ^82^ RIP1 not only acts as an adaptor to recruit IKK to TNFR1 by interacting with IKKγ but can also indirectly activate the IKKβ subunit of IKK by activating TAK1 through ubiquitination.

TNFR2 activates not only the classical NF‐κB and MAPK pathways mentioned above but also the alternative p52‐RelB NF‐κB signaling pathway.^83^ TNFR2 recruits TRAF3 and cIPA1/2 through TRAF2, and TRAF3 binds to NIK and promotes NIK degradation.^81^ cIPA1/2 mediates the ubiquitination and degradation of TRAF2 and TRAF3 to release NIK.^84^, ^85^ NIK phosphorylates IKKα to promote the phosphorylation and partial degradation of p100 to generate p52, which allows p52 to bind RelB, forming an NF‐κB dimer to mediate the transcription of target genes.^83^, ^84^ Degradation of TRAF3 also unblocks the TRAF2/6:MAP3K signaling complexes, leading to translocation of the complex from the receptor to the cytoplasm, where it initiates MAPK phosphorylation and cascade activation.^81^

In addition, CD40 is also a member of the TNFR superfamily expressed on macrophages. Binding of CD40 to its ligand CD40L activates the NF‐κB and MAPK signaling pathways mainly through TRAFs (TRAF2/3/5/6), inducing stronger macrophage antigen‐presenting ability and the production of proinflammatory cytokines and chemokines.^82^, ^86^

### 3.3 IL receptor‐related signaling pathways

#### 3.3.1 IL‐4/IL‐4R

**FIGURE 2:**
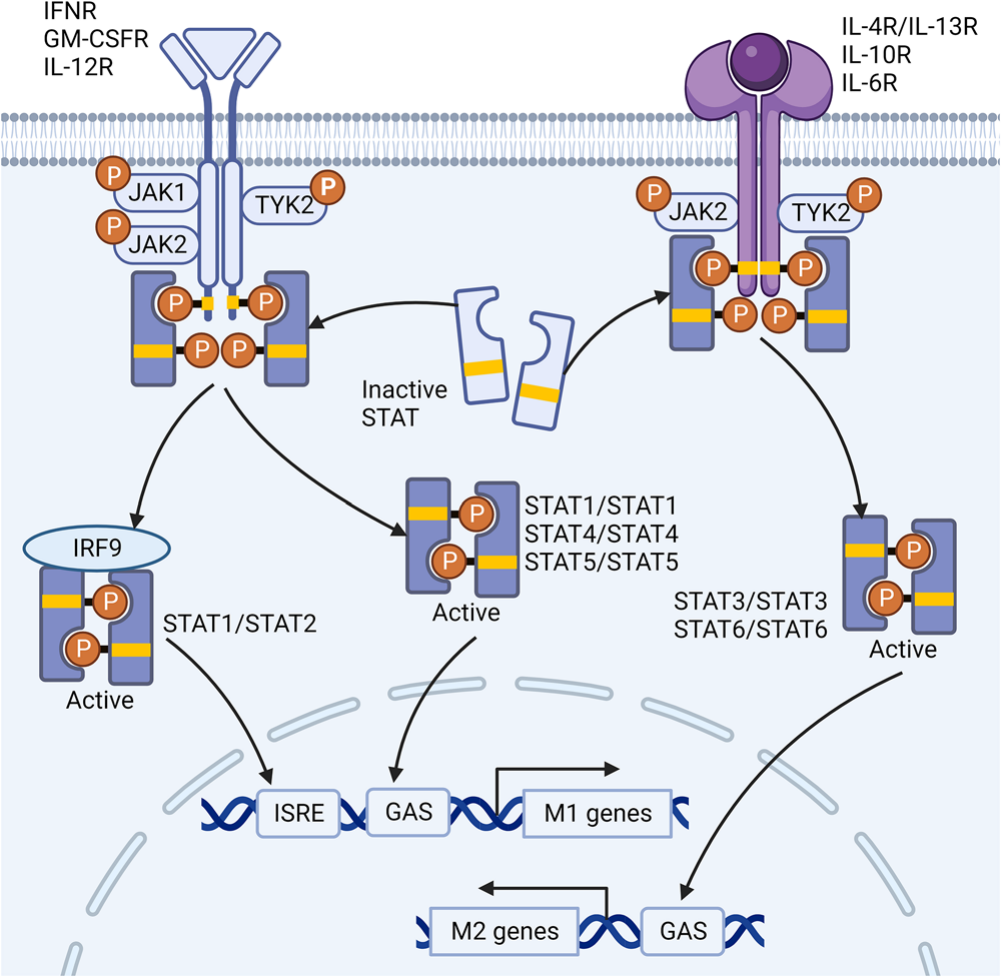
Activation of the JAK‐STAT signaling pathway in macrophages. The JAK‐STAT axis is one of the important pathways that regulate macrophage polarization and function. Various cytokines such as IFN‐γ, GM‐CSF, IL‐4/13, IL‐6, and IL‐10 can utilize specific combinations of JAK and STAT members to activate the formation of STAT homodimers or heterodimers, thereby affecting gene transcription. STAT homodimers can directly bind to GAS to promote the transcription of M1/M2‐related genes, while heterodimers such as STAT1/STAT2 bind to IRF9 and then to ISRE to promote M1‐related genes transcription. Abbreviations: IFN, interferon; GM‐CSF, granulocyte‐macrophage colony‐stimulating factor; IL, interleukin; JAK, Janus kinase; STAT, signal transducer and activator of transcription; ISRE, IFN‐stimulated response elements; GAS, IFN‐γ‐activated sequences. (Created with BioRender.com).

IL‐4 is an important activator of macrophage M2 polarization and initiates the JAK‐STAT (Figure 2) and phosphatidylinositol 3‐kinase (PI3K)/protein kinase B (Akt) signaling pathways by binding to IL‐4R.^32^, ^87^ There are two types of IL‐4R: type I receptors consisting of the IL‐4R alpha chain (IL‐4Rα) and common gamma chain (γc) and type II receptors consisting of IL‐4Rα and IL‐13Rα1. Regardless of the type of receptor, these receptor subunits all interact with the Janus kinase (JAK) family. The JAK family consists of four isoforms: JAK1, JAK2, JAK3, and TYK2, all of which are nonreceptor tyrosine kinases. IL‐4Rα binds JAK1, while γc and IL‐13Rα1 bind JAK3 and TYK2, respectively. The interaction between IL‐4 and IL‐4R activates the STAT6 through JAK1/3. Then, activated STAT6 dissociates from IL‐4R to form a dimer with another STAT6 molecule through its SH2 domain.^87^, ^88^ Translocation of this dimer into the nucleus promotes transcription of M2‐related genes such as *Mrc1*, *Arg1*, and *IL‐10*.^88^ In addition, IL‐4Rα phosphorylated tyrosine residues can also recruit and phosphorylate the insulin receptor substrate (IRS) family, specifically IRS2. Phosphorylated IRS attracts the p85 subunit of PI3K, leading to the activation of PI3K/Akt/mammalian target of rapamycin (mTOR) signaling.^89^ Moreover, IRS interacts with growth factor receptor‐bound protein 2 (GRB2) to activate son of sevenless (SOS), which results in MAPK pathway activation.^90^

#### 3.3.2 IL‐6/IL‐6R

The receptor complex formed by IL‐6, IL‐6R, and gp130 activates intracellular signaling cascades, including the JAK1‐STAT3 (Figure 2) and MAPK signaling pathways, to promote M2 macrophage polarization.^91^ In addition, IL‐6 signaling enhances the macrophage response to IL‐4. gp130‐bound JAKs, especially JAK1, are activated after receptor complex formation. Then, activated JAK1 phosphorylates different tyrosine residues in the intracellular segment of the receptor and recruits STAT3, which is also phosphorylated by JAK1. STAT3 forms a homodimer and translocates to the nucleus to activate the transcription of M2‐related genes. Phosphorylated receptor tyrosine residues also activate GRB2 to trigger the Ras/Raf/MAPK signaling cascade.

#### 3.3.3 IL‐10/IL‐10R

IL‐10 is a critical anti‐inflammatory cytokine and is involved in promoting M2 polarization of macrophages. IL‐10R consists of two subunits, IL‐10Rα and IL‐10Rβ, which interact with JAK1 and TYK2, respectively. In the presence of IL‐10, IL‐10R activates JAK1 and TYK2 to promote STAT3 phosphorylation.^92^ Activated STAT3 dimerizes and translocates to the nucleus to promote M2‐related gene expression and inhibit IL‐6, IL‐8, and TNF‐α production.^92^, ^93^

### 3.4 IFN receptor‐related signaling pathways

Recognition of IFN and IFN receptor promotes M1 macrophage polarization by activating the JAK‐STAT signaling pathway (Figure 2). Although type I IFN and IFN‐γ bind to different receptors, these receptors all interact with members of the JAK family. The binding of IFN‐γ causes dimerization and activation of IFN‐γR1 and IFN‐γR2, which leads to autophosphorylation of JAK 1 and JAK2.^94^ Activated JAK2 phosphorylates IFN‐γR to form a docking site for STAT1, causing STAT1 recruitment and phosphorylation. Phosphorylated STAT1 dissociates from IFN‐γR and undergoes homodimerization and nuclear translocation to regulate IFN‐stimulated genes (ISGs) expression by binding to IFN‐γ‐activated sequences.^95^ In addition, type I IFNs, such as IFN‐α and IFN‐β, promote the formation of not only STAT homodimers but also heterodimers, such as STAT1‐STAT2. STAT1‐STAT2 binds to IRF9 to form a triplet complex called ISG factor 3 (ISGF3).^96^ ISGF3 regulates the expression of ISGs by binding to ISRE to promote the M1 polarization of macrophages.^95^, ^96^

### 3.5 CSF‐related signaling pathways

#### 3.5.1 CSF‐1/CSF‐1R

**FIGURE 3:**
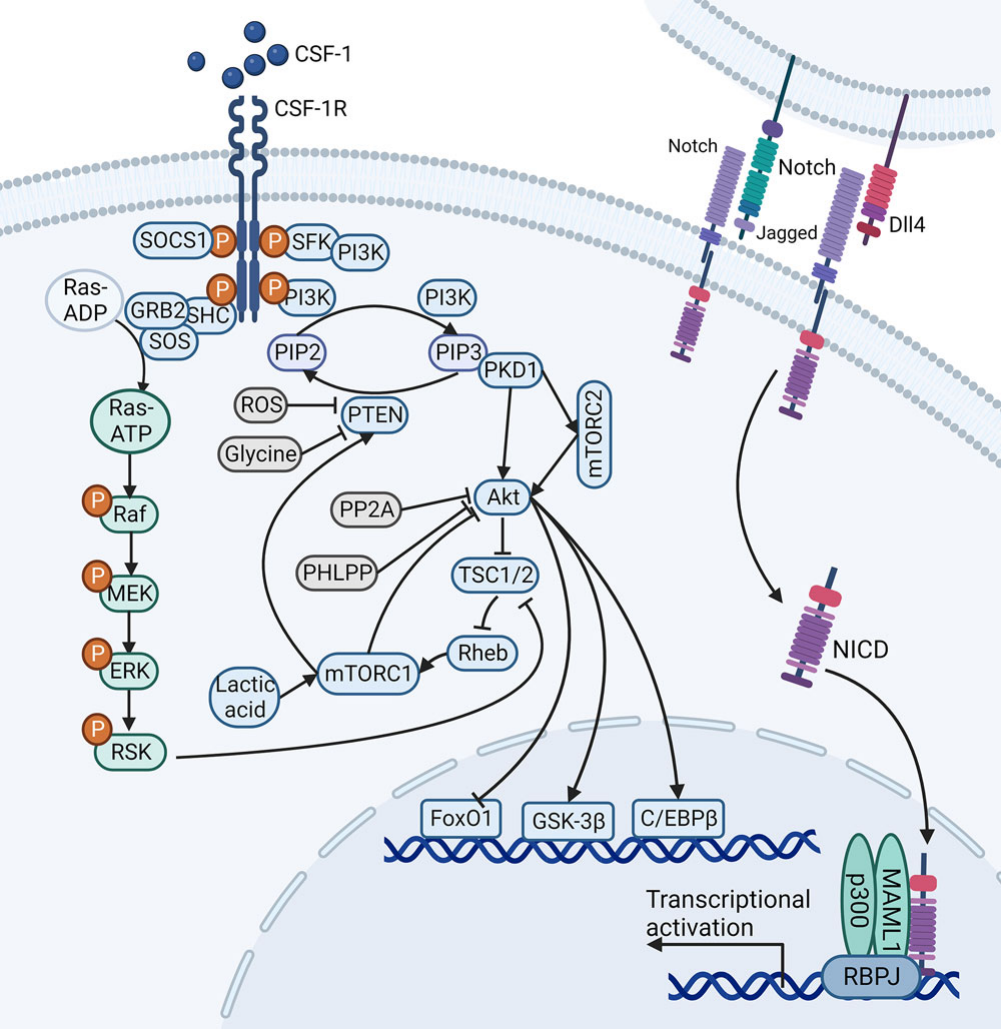
Activation of PI3K/Akt and Notch signaling pathways in macrophages. When bound to CSF‐1, phosphorylation sites in intracellular segment of CSF‐1R activate PI3K to initiate PI3K/Akt signaling pathway. Akt regulates the proliferation, polarization, and survival of macrophages by activating or inhibiting transcription factors such as FoxO1, GSK‐3β, and C/EBPβ. PTEN and lactic acid are negative regulators of this pathway, while glycine and ROS promoted the signaling response by inhibiting PTEN. The binding of Notch to its ligand Dll4 results in the release of Notch intracellular domain into the nucleus and the formation of transcription activation complex to promote the expression of M1‐related genes in macrophages. In addition, Jagged1‐Notch axis promotes M2‐related genes expression in tumor‐associated macrophages. Abbreviations: CSF‐1, colony‐stimulating factor‐1; CSF‐1R, colony‐stimulating factor‐1 receptor; PI3K, phosphatidylinositol 3‐kinase; SFK, Src family tyrosine kinases; RSK, ribosome protein subunit 6 kinase; PTEN, phosphatase and tensin homolog deleted on chromosome ten; PIP2, phosphati‐dylinositol‐4,5‐bisphosphate; PIP3, phosphati‐dylinositol‐3,4,5‐triphosphate; PKD, protein kinase D; mTORC, mammalian target‐of‐rapamycin complex; TSC, tuberous sclerosis complex; FoxO1, forkhead box protein O1; GSK‐3β, glycogen synthase kinase‐3β; C/EBPβ, CCAAT/enhancer binding protein β; Dll4, Delta‐like 4; NICD, Notch intracellular domain; MAML1, mastermind‐like transcriptional coactivator 1; RBPJ, recombination signal binding protein for immunoglobulin κJ region. (Created with BioRender.com).

Signaling pathways mediated by CSF‐1/CSF‐1R regulate macrophage survival, proliferation, chemotaxis, and differentiation.^21^, ^97^ The binding of CSF‐1 to its receptor CSF‐1R rapidly triggers CSF‐1R dimerization and phosphorylation of multiple intracellular tyrosine sites,^98^, ^99^ allowing CSF‐1R to phosphorylate molecules such as PI3K, Src, and GRB2.^100^ PI3K is an essential downstream signaling molecule of CSF‐1R (Figure 3).^101^ Activated PI3K induces phosphorylation of phosphati‐dylinositol‐4,5‐bisphosphate (PIP2) to form phosphati‐dylinositol‐3,4,5‐triphosphate, which not only recruits and activates Akt through the pleckstrin homology domain but also enhances Akt activation by phosphorylating mammalian target‐of‐rapamycin complex 2 (mTORC2).^102^ Akt regulates macrophage proliferation, polarization, and metabolism through various downstream effectors.^102^ For example, Akt inhibits forkhead box protein O1 (FoxO1), a transcription factor that promotes the expression of proinflammatory and proliferation‐ and survival‐related genes in M1 macrophages.^103^ The phosphorylation of glycogen synthase kinase‐3β (GSK‐3β) by Akt can promote the M2 polarization of macrophages.^104^ CCAAT/enhancer binding protein β (C/EBPβ) is involved in macrophage activation, whose expression is controlled by Akt2.^105^ mTORC1 is also an important downstream signaling molecule of Akt.^106^ mTORC1 regulates the synthesis of various proteins in macrophages by activating S6K and 4EBP1, thus regulating the metabolism and function of macrophages.^102^, ^107^ In addition, CSF‐1 regulates macrophage adhesion and motility by activating Rho, Rac, and Cdc, which are central regulators of the actin cytoskeleton and adhesion structures, through at least two independent pathways (the Src/Pyk2 and PI3K pathways).^97^ In addition, phosphorylation of CSF‐1R Y697 activates GRB2 and suppressor of cytokine signaling 1 (SOCS1), while Y706 phosphorylation is required for complete STAT1 activation.^98^ GRB2 is involved in the activation of the Ras/Raf/mitogen‐activated extracellular signal‐regulated kinase (MEK)/extracellular signal‐regulated kinase (ERK) pathway.^21^, ^97^ SOCS1 is an important negative feedback regulator of signal transduction that inhibits JAK‐STAT1 and NF‐κB pathways activation in macrophages.^108^

#### 3.5.2 GM‐CSF)/GM‐CSF and its receptor (GM‐CSFR)

The interaction between GM‐CSF and its receptor (GM‐CSFR) triggers M1 macrophage polarization and proinflammatory mediator production mainly through the JAK2‐STAT5 signaling pathway. GM‐CSF binds to its receptors GM‐CSFRα and GM‐CSFRβ to form a dodecamer ligand‒receptor complex that promotes STAT5 phosphorylation and homodimerization via JAK2.^109^ Then, STAT5 dimers promote the transcription of M1‐related genes and the production of inflammatory mediators such as TNF‐α, IL‐6, IL‐12, and IL‐23.^110^ In addition, GM‐CSF also activates the macrophage NF‐κB and PI3K/Akt and Ras/MEK/ERK signaling pathways.^110^, ^111^

### 3.6 Growth factor‐related signaling pathways

#### 3.6.1 TGF‐β/TGF‐β receptor (TβR)

**FIGURE 4:**
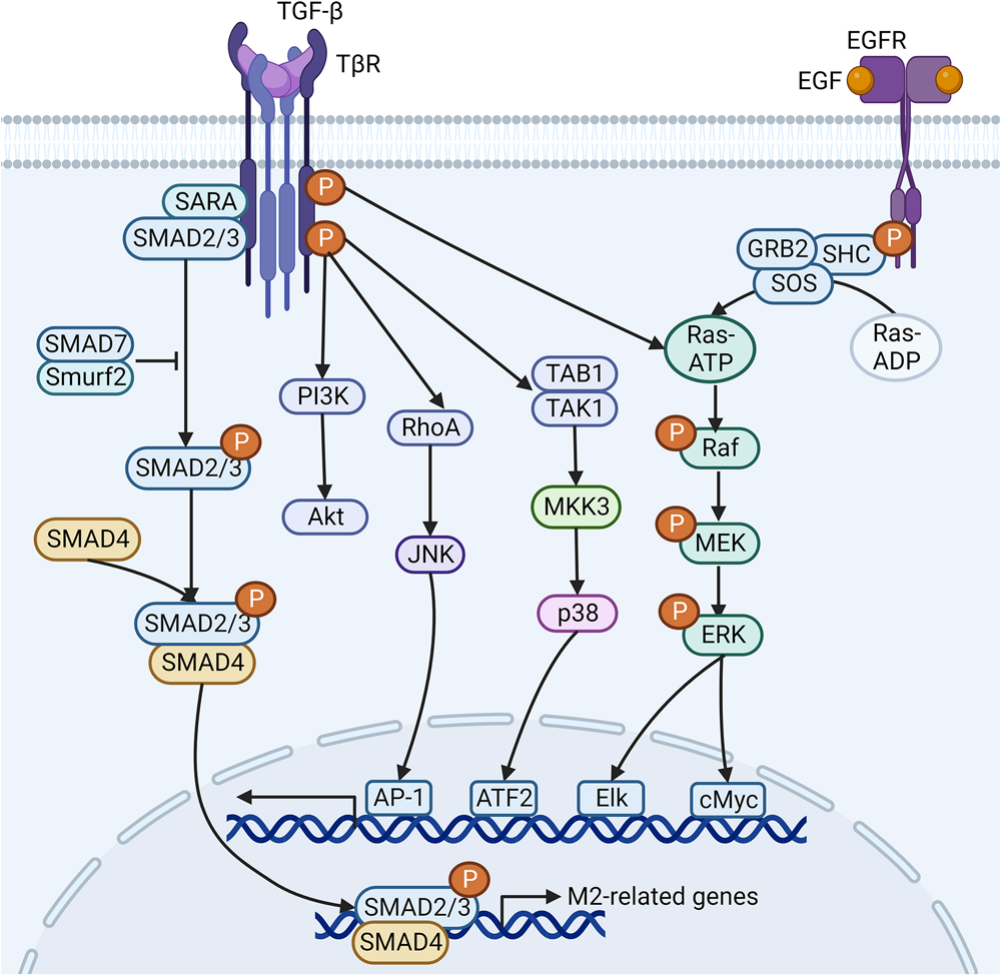
Activation of MAPK and TGF‐β‐SMAD signaling pathways in macrophages. MAPK signaling pathway can be activated by molecules such as TGF‐β and EGF. The MAPK signal cascade consists of three kinds of protein kinases (MAP3K, MAP2K, and MAPK), which transmit upstream signals to downstream response molecules through sequential phosphorylation. Raf and MEKKs belong to MAP3K; MEKs belong to MAP2K; JNK, ERKs and p38 belong to MAPK. AP‐1, a heterodimer composed of c‐Fos and c‐Jun. TGF‐β activates SMAD4 via SMAD2/3 to regulate the expression of M2 polarization‐related genes. Abbreviations: EGF, epidermal growth factor; SHC, Src homolog and collagen homolog; GRB2, growth factor receptor‐bound protein 2; SOS, son of sevenless; MAP3K, mitogen‐activated protein kinase kinase kinase; MEK, mitogen‐activated extracellular signal‐regulated kinase, MAP2K, mitogen‐activated protein kinase kinase; JNK, c‐Jun kinase; ERK, extracellular signal‐regulated kinase; MAPK, mitogen‐activated protein kinase; ATF2, activating transcription factor 2; TGF‐β, transforming growth factor‐β; TβR, TGF‐β receptor; SMAD, Sma‐ and Mad‐related protein; SARA, the SMAD anchor for receptor activation; Smurf2, SMAD‐specific E3 ubiquitin protein ligase 2; RhoA, Ras homolog family member A. (Created with BioRender.com).

TGF‐β regulates macrophage polarization and function through the TβR in Sma‐ and Mad‐related protein (SMAD)‐dependent or SMAD‐independent signal transduction pathways (Figure 4).^112^ When TGF‐β drives the formation of a heterotetrameric complex consisting of two TβRI and two TβRII, TβRII phosphorylates the serine or threonine residues of TβRI, thereby phosphorylating SMAD2/3 to initiate the SMAD signaling cascade.^112^ Activated SMAD2/3 separate from TβR and then interact with SMAD4, moving into the nucleus to promote the expression of M2‐related genes. In addition, TGF‐β/SMAD6 induces polyubiquitination and degradation of MyD88 by recruiting the E3 ubiquitin ligases Smurf 1/2 to inhibit TLR/NF‐κB.^113^ SMAD6 also segregates Pellino‐1, which is required for the IL‐1R/TLR proinflammatory response.^114^ TGF‐β/SMAD7 affects TNF signaling by blocking TNF‐induced TAK1 activity.^115^ Moreover, TGF‐β can phosphorylate PI3K,^116^ ERK,^117^, ^118^ independent of SMADs, which is mainly achieved by TβR‐induced ubiquitination of TRAF6.

#### 3.6.2 Epidermal growth factor (EGF)/EGF and its receptor (EGFR)

EGF plays an important role in macrophage activation through MAPK signaling pathways (Figure 4).^119^, ^120^ The binding of EGFR causes EGFR dimerization, which leads to C‐terminal tail autophosphorylation of EGFR, and the phosphorylation region acts as an anchor for GRB2.^121^ Then, GRB2 recruits and binds to SOS to trigger the Ras/Raf/MAPK signaling pathway.^121^ Activation of the MAPK cascade results in the transcription of many cytokine genes.^119^, ^120^ Phosphorylated EGFR also causes tyrosine phosphorylation of PI3K to activate the PI3K/Akt/mTOR signaling pathway.^121^

### 3.7 G‐protein‐coupled receptor (GPCR) signaling pathway

GPCRs are a large family of receptors that can bind to various ligands, including chemokines, hormones, ions, neurotransmitters, and odorant molecules.^122^, ^123^ Macrophages express many GPCRs, such as the chemokine receptor Frizzled (Fzd), which plays a key role in regulating the differentiation and function of macrophages.^124^ Here, we mainly introduce C‐C chemokine receptor (CCR) type 2 (CCR2)/CCR5‐ and Fzd‐mediated signal transduction in macrophages (the regulation of other GPCRs on macrophages has been detailed in the review^124^).

**FIGURE 5:**
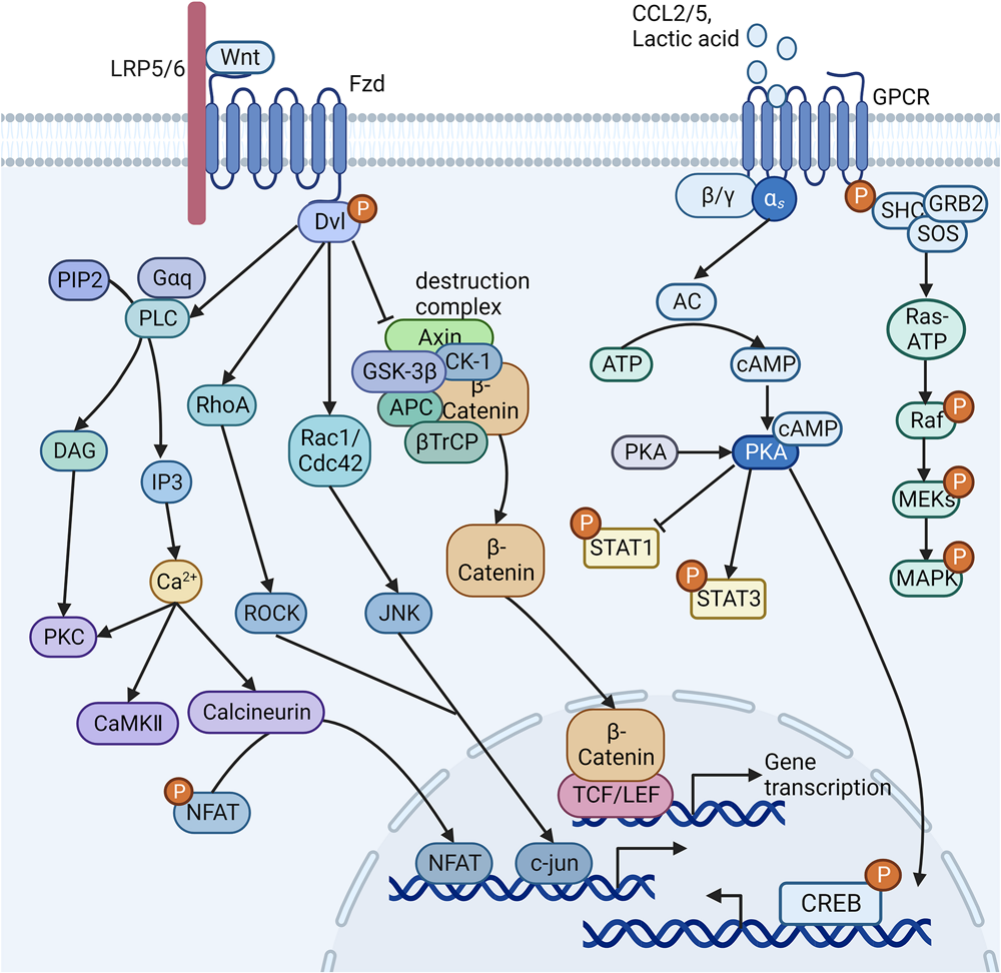
Activation of GPCR signaling pathway in macrophages. Chemokines such as CCL2/CCL5, and lactic acid, are signaling molecules that bind to GPCRs causing conformational changes that activate the dissociation of G proteins into Gα and Gβγ. Gα activates PKA by promoting cAMP production, inducing CREB phosphorylation and nuclear translocation thereby promoting anti‐inflammatory cytokine production and macrophage M2 polarization. PKA also inhibits STAT1 phosphorylation and promotes STAT3 phosphorylation contributing to M2 macrophage polarization. Wnt combines with its receptor Fzd 1 to activate downstream signal transduction and further regulate macrophage polarization and function. Wnt2b and Wnt3a inhibit the destruction complex formed by APC, Axin, βTrCP and GSK‐3β through Dvl, leading to the accumulation of β‐catenin and its translocation to the nucleus where it binds to TCF/LEF to mediate anti‐inflammatory and M2‐related gene expression. In contrast, Wnt5a binding to Fzd5 activates the phagocytic activity and pro‐inflammatory response of macrophages through Dvl activation of RhoA, Rac1/Cdc42, and PLC. Abbreviations: GPCR, G‐protein‐coupled receptors; AC, adenylate cyclase; cAMP, cyclic adenosine monophosphate; PKA, protein kinase A; CREB, cAMP‐response element binding protein; Wnt, wingless homolog; Fzd, Frizzled; LRP, LDL receptor‐related protein; Dvl, dishevelled; PLC, phospholipase C; DAG, diacylglycerol; IP3, inositol triphosphate; CaMKII, calmodulin‐dependent protein kinase type II; NFAT, nuclear factor of activated T cells; ROCK, Rho‐associated kinase; CK‐1, casein kinase 1; TCF, T‐cell factor; LEF, lymphoid enhancer‐binding factor. (Created with BioRender.com).

The binding of CCL2/CCL5 to its receptor CCR2/CCR5 promotes macrophage M2 polarization through the GPCR signaling pathway. When bound to its ligand, the GPCR undergoes conformational changes that cause the dissociation of G protein into Gα and Gβγ. Then, Gα and Gβγ activate their downstream signaling molecules and generate different intracellular signal transduction pathways (Figure 5).^122^ Gα activates adenylyl cyclase to convert ATP to cyclic adenosine monophosphate (cAMP),^125^ which activates protein kinase A (PKA). Activated PKA promotes the phosphorylation of cAMP‐responsive element binding protein (CREB),^126^ and CREB promotes the expression of anti‐inflammatory cytokines and M2‐related genes. PKA also activates ERK1/2 and STAT3 but inhibits NF‐κB and STAT1.^127^ Gα activates phospholipase C (PLC) to catalyze the production of inositol triphosphate (IP3) and diacylglycerol from PIP2 as well. The binding of IP3 to calcium channel receptors on the ER membrane leads to ER calcium efflux, increasing cytoplasmic Ca^2+^, which activates various protein kinases, including protein kinase C (PKC) and calmodulin‐dependent protein kinase type II (CaMKII), to regulate macrophage activation and function.^124^

Unlike CCR2/CCR5, Fzd1 is a member of another family of GPCRs. When combined with its ligand wingless homolog (Wnt), Fzd1 activates the Wnt/β‐catenin, Wnt/planar cell polarity (PCP), and Wnt/Ca2+ pathways (Figure 5) to regulate macrophage polarization and function, and different Wnt isoforms can contribute to M2 or M1 polarization of macrophages.^128^ Some Wnt molecules, such as Wnt2b and Wnt3a, can mediate the anti‐inflammatory response and M2 polarization of macrophages through the Wnt/β‐catenin signaling pathway.^129^, ^130^ In the absence of Wnt signaling, cytosolic β‐catenin is phosphorylated and then degraded by the destruction complex (DC), which is formed by APC, Axin, β‐transducin repeats‐containing protein (βTrCP), and GSK‐3β. Binding of Wnt3a to Fzd1 blocks the effect of DC on β‐catenin through dishevelled (Dvl), thereby leading to the accumulation of β‐catenin in the cytoplasm and translocation to the nucleus, where it binds to T‐Cell Factor/Lymphoid Enhancer‐Binding Factor to promote the transcription of downstream target genes such as *c‐myc*, *c‐jun*, peroxisome proliferator activated receptor (PPAR) δ, and cyclin D1.^128^ Moreover, the Wnt/β‐catenin pathway can antagonize the Notch pathway^131^ and restrain NF‐κB signaling activation by inhibiting GSK‐3β activity.^129^, ^132^

The interaction between Wnt5a and Fzd5 activates Wnt/PCP and Wnt/Ca^2+^, thereby stimulating the phagocytic activity of macrophages and the secretion of proinflammatory cytokines.^129^, ^133^ Fzd5 activates RhoA and Rac1/Cdc42, which activate Rho‐associated kinase (ROCK) and JNK, respectively, via Dvl. ROCK and JNK translocate into the nucleus to bind to c‐jun and Activating Transcription Factor 2 to regulate gene expression and promote proinflammatory factor production.^128^ Dvl also activates PLC, promoting the accumulation of Ca^2+^ in the cytoplasm, which induces the expression of proinflammatory cytokines by activating CaMKII.^134^

### 3.8 Notch signaling pathway

The Notch signaling pathway is involved in regulating macrophage polarization and function.^135^ To date, four NOTCH receptors (Notch1–4) and five NOTCH ligands (Jagged1, Jagged2, Delta‐like [Dll] 1, Dll3, and Dll4) have been identified.^136^ Dll4‐Notch can promote the inflammatory response and M1 polarization of macrophages,^137^, ^138^ while the Jagged1‐Notch axis promotes M2 macropahge polarization.^139^ The Notch receptor is composed of Notch extracellular domain, Notch intracellular domain (NICD), and transmembrane domain.^136^ When binding to Dll4 on the surface of adjacent cells, Notch proteolysis occurs twice to release NICD into the nucleus, which binds to the nuclear transcription factor CSL.^137^, ^140^ In the absence of Notch signaling, CSL binds specifically to co‐inhibitory receptors, histone deacetylase, and DNA sequences, forming a CSL‐DNA binding protein complex to silence the expression of downstream genes.^141^ When CSL binds to NCID, it separates from repressor molecules and recruits transcription coactivators to form a transcription activation complex, resulting in the promotion of M1‐related gene expression (Figure 3).^140^

### 3.9 Triggering receptor expressed on myeloid cells (TREM) signaling pathway

The TREM is a class of innate immune receptors that plays a key role in regulating the inflammatory response by amplifying or inhibiting TLR‐induced signals.^142^ TREM exists in two forms, membrane receptor TREM and soluble TREM (sTREM), and sTREM negatively regulates membrane TREM signaling by competitively neutralizing ligands.^142^

TREM‐1 is an activating receptor expressed on monocytes and macrophages that effectively amplifies the inflammatory response by inducing inflammatory mediator secretion and cooperating with PRR‐mediated signaling pathways.^143^ Since TREM‐1 does not contain any signaling motif, it binds to DAP12, an adaptor protein with an ITAM to mediate intracellular signaling.^143^ The ITAMs of DAP12 undergo tyrosine residue phosphorylation, providing a docking site for SYK. SYK activates multiple signal transduction pathways, including Ras/ERK and NF‐κB, thereby promoting the expression of proinflammatory cytokines.^144^, ^145^ TREM‐1 also prolongs macrophage survival by upregulating the Egr2‐dependent antiapoptotic protein Bcl‐2 via ERK and increasing mitofusin 1/2, which maintains mitochondrial integrity.^146^ Moreover, TREM‐1 enhances the activity of molecules including MyD88, IκBα, and NF‐κB.^147^, ^148^

TREM‐2 is an immunonegative regulator that inhibits macrophage activation and the inflammatory response.^149^ The low‐avidity interaction between TREM‐2 and DAP12 results in incomplete phosphorylation of DAP12, which fails to recruit and activate SYK but leads to preferential activation of the inhibitory phosphatase SHP1, thereby inhibiting TLR‐mediated activation.^144^ In addition, DAP12‐activated PI3K/Akt blocks MAP3K, and DAP12‐activated PLCγ may reduce PIP2, a TIRAP docking site that is required for MyD88 recruitment to the TLR signaling complex.^144^

### 3.10 Immunosuppressive receptor‐related signaling pathways

Signal regulatory protein α (SIRPα), a myeloid inhibitory receptor containing immunoreceptor tyrosine‐based inhibitory motifs (ITIMs), inhibits macrophage phagocytosis and proinflammatory cytokine production by interacting with its cell surface ligand CD47.^150^ When SIRPα binds CD47, ITIMs of SIRPα are tyrosine phosphorylated by SFK to bind and inhibit the function of SHP1 and SHP2. Then, SHP1 and SHP2 are engaged in various signaling pathways, such as the GRB2/Ras/Raf/MAPK, JAK‐STAT and PI3K pathways.^151^, ^152^

Similar to SIRPα, LILRB,^153^ PD‐1,^154^ and Siglec‐10^155^ are also important immunosuppressive receptors expressed on macrophages, all of which contain ITIMs.^156^ When these receptors bind to their ligands MHC I, programmed death‐ligand 1 (PD‐L1), and CD24, they inhibit the activation of Ras/ERK and NF‐κB through ITIMs, thereby inhibiting the phagocytosis and activation of macrophages.^153^, ^154^, ^155^

### 3.11 Redox signaling pathways

ROS are composed of free radicals and non‐free radicals formed by partial reduction of oxygen, including superoxide anion, hydrogen peroxide, and hydroxyl radical.^157^ One of the most important characteristics of ROS is that they can snatch electrons from others and are extremely susceptible to oxidation reactions with others. ROS are not only important mediators produced by macrophages to clear pathogens and participate in innate immunity but also have the ability to participate in signaling pathways as second messengers to regulate the phenotype and function of macrophages.^158^, ^159^ ROS promote the proinflammatory response and M1 polarization of macrophages by promoting the NF‐κB and MAPK signaling cascades. ROS induce thioredoxin, an inhibitor of apoptotic signaling regulated kinase (ASK1), to dissociate from the trx‐ASK1 complex, resulting in phosphorylation of ASK1 and activation of downstream p38.^160^ ROS also indirectly maintain the activity of this cascade by inactivating JNK‐inhibiting phosphatase.^161^ Moreover, ROS can trigger NLRP3 activation to regulate macrophage pyroptosis.^162^ In addition, ROS enhance the phosphorylation of IκB to activate NF‐κB^163^ and participate in the activation of STAT1 and IRF5.^164^ However, ROS are also involved in regulating M2 macrophage polarization under certain circumstances. For instance, in the presence of M‐CSF, ROS are involved in the late activation of ERK signaling required for M2‐like polarization of macrophages in cancer,^165^ and in alveolar macrophages, ROS directly oxidize specific cysteine residues of STAT6 to promote transcription of the M2‐related genes *Arg 1* and *FIZZ1*.^166^ ROS can also maintain the activation of the PI3K/Akt pathway by inhibiting PTEN.^167^

### 3.12 Lactic acid signaling pathways

Lactate drives macrophage polarization to anti‐inflammatory, pro‐repair phenotypes through regulation of multiple signaling pathways.^168^, ^169^ First, lactate inhibits proinflammatory signaling through GPCRs.^170^ Lactate specifically inhibits the activation of inflammasome and TLR4‐mediated NF‐κB by activating GPR81 on macrophages.^170^ Lactic acidosis mediates cAMP‐dependent signaling by activating plasma membrane GPCRs, inducing the expression of M2 genes and the transcriptional repressor cAMP‐responsive element modulator (CREM/ICER). CREM inhibits the expression of inflammatory genes, including TNF and NOS2.^171^, ^172^ Second, lactate not only stabilizes HIF1α^173^ but also decreases *ATP6V0d2* expression by activating mTORC1. ATP6V0d2 is involved in the formation of vacuolar proton pumps that promote lysosomal acidification and protein degradation. Reduced expression of *ATP6V0D2* leads to a reduction in the degradation of HIF2α by lysosomes, which induces high expression of VEGF and M2 homeostatic genes.^174^ Third, lactate inhibits RIG‐I‐MAVS‐mediated type I IFN production by binding to MAVS, thereby suppressing proinflammatory responses. In addition, lactate inhibits the excessive activation of the NLRP3 inflammasome and caspase‐1 to suppress macrophage pyroptosis and the inflammatory response.^175^

### 3.13 Glycinergic signaling pathways

Glycine can shape macrophage polarization and function by regulating signaling pathways such as NF‐κB and PI3K/Akt.^176^ First, glycine inhibits the activation of IKK by inhibiting the phosphorylation of IKKα and IKKβ and the degradation of IκB in proinflammatory macrophages,^177^ thereby inhibiting NF‐κB activation and M1 polarization. Second, glycine restrains NLRP3 expression and inflammasome assembly in M1 macrophages through upregulation of the Nrf‐2/HO‐1 axis,^178^ inhibiting the inflammatory response and pyroptosis. Finally, glycine could block PTEN to upregulate the activation of Akt in microglia, which inhibits the activation of NF‐κB and HIF1α.^179^

## 4 THE ROLE OF MACROPHAGES IN DISEASES AND TARGETED THERAPIES

**FIGURE 6:**
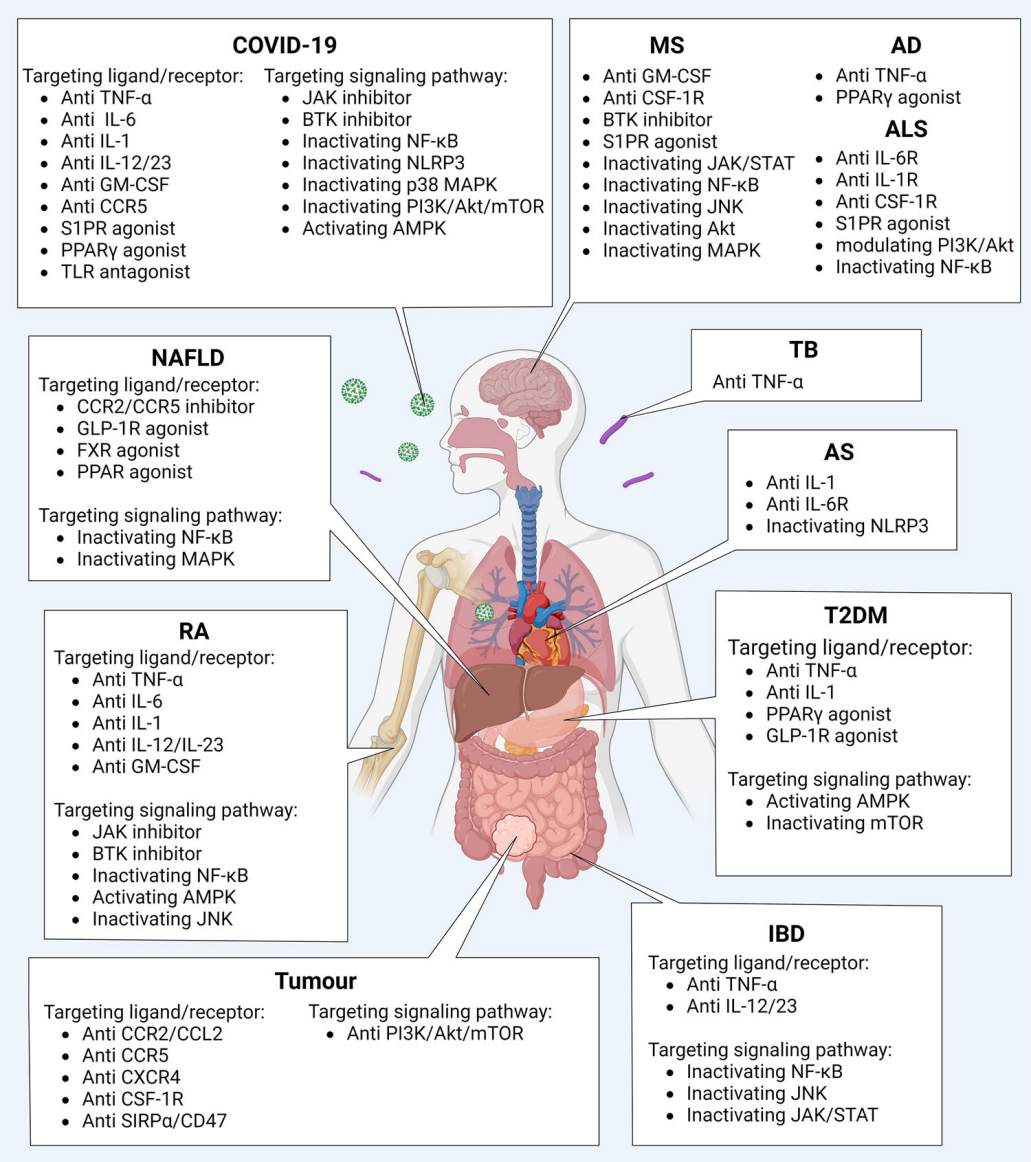
Therapeutic targets targeting macrophage in human diseases. Macrophages are widely present in almost all tissues of adult and play important roles in pathological processes. With the gradual in‐depth understanding of molecular mechanisms, more alternative therapeutic targets of macrophage have been proposed and applied in clinic/clinical trials. Abbreviations: COVID‐19, coronavirus disease 2019; NAFLD, nonalcoholic fatty liver disease; RA, rheumatoid arthritis; MS, multiple sclerosis; AD, Alzheimer's disease; ALS, amyotrophic lateral sclerosis; TB, tuberculosis; AS, atherosclerosis; T2DM, type 2 diabetes mellitus; IBD, inflammatory bowel disease. (Created with BioRender.com).

Since macrophages are important contributors to development, tissue homeostasis, immunity, and repair, macrophage dysfunction is involved in the initiation and development of many diseases, such as autoimmune, neurodegenerative, metabolic, and infectious diseases and cancer. It is becoming increasingly clear that the flexible and precise transformation of the functional phenotypes of macrophages plays important roles in the progression or remission of diseases. Therapies targeting components and regulators of signaling pathways in macrophages hold great potential and are a prospective direction for the treatment of these diseases (Table 1; Figure 6).

**TABLE 1:** Drug candidates in clinic/clinical trials for signaling pathway targeted therapy of macrophage in human diseases.

Therapeutic target	Agent	Mechanism of action	Application in disease	Drug status	Ref.
Drugs targeting ligand/receptor on macrophage					
TNF‐α	Infliximab	TNF‐α inhibitor	RA, IBD	Marketed	362, 363
			COVID‐19	Clinical trial Phase IV	364
	Adalimumab	TNF‐α inhibitor	RA, IBD	Marketed	362, 363
			COVID‐19	Clinical trial Phase III	365
	Golimumab	TNF‐α inhibitor	RA, IBD	Marketed	362, 363
	Certolizumab pegol	TNF‐α inhibitor	RA, IBD	Marketed	362, 363
	Etanercept	Soluble recombinant TNF receptor fusion protein	RA	Marketed	362
			AD	Clinical trial Phase II	222
			Tuberculosis	Clinical trial Phase IV	297
IL‐6	Siltuximab	IL‐6 inhibitor	RA	Marketed	366
			COVID‐19	Clinical trial Phase III	367
	Sirukumab	IL‐6 inhibitor	RA	Clinical trial Phase III	368
			COVID‐19	Clinical trial Phase II	306
	Olokizumab	IL‐6 inhibitor	RA	Clinical trial Phase III	369
			COVID‐19	Clinical trial Phase III	306
	Clazakizumab	IL‐6 inhibitor	RA	Clinical trial Phase II	370
			COVID‐19	Clinical trial Phase II	371
	Tocilizumab	IL‐6R inhibitor	RA	Marketed	366
			ALS	Clinical trial Phase II	250
			AS	Clinical trial Phase III	286
			COVID‐19	Marketed	372
	Salirumab	IL‐6R inhibitor	RA	Clinical trial Phase II	373
	Levilimab	IL‐6R inhibitor	COVID‐19	Clinical trial Phase III	374
IL‐1	Anakinra	IL‐1R antagonist	RA	Marketed	261
			ALS	Clinical trial Phase II	252
			T2DM	Clinical trial Phase IV	261
			AS	Clinical trial Phase IV	286
			COVID‐19	Clinical trial Phase III	375
	Canakinumab	IL‐1β antagonist	RA	Clinical trial Phase IV	376
			T2DM	Clinical trial Phase III	377
			AS	Clinical trial Phase III	284, 285
			COVID‐19	Clinical trial Phase IV	378
	MABp1	IL‐1α antagonist	T2DM	Clinical trial Phase II	262
IL‐12/23	Ustekinumab	Antagonist of the p40 subunit of IL‐12 and IL‐23	RA	Clinical trial Phase II	379
			IBD	Marketed	363
	Risankizumab	Antagonist of the p19 subunit of IL‐23	IBD	Marketed	363
			COVID‐19	Clinical trial Phase II	306
	Brazikumab	Antagonist of the p19 subunit of IL‐23	IBD	Clinical trial Phase III	363
	Mirikizumab	Antagonist of the p19 subunit of IL‐23	IBD	Clinical trial Phase III	380
	Guselkumab	Antagonist of the p19 subunit of IL‐23	RA	Clinical trial Phase II	379
			IBD	Clinical trial Phase III	381
GM‐CSF	KB003	GM‐CSF inhibitor	RA	Clinical trial Phase II	190
	MOR103	GM‐CSF inhibitor	RA	Clinical trial Phase II	382
			MS	Clinical trial Phase I/II	206
	MORAb‐022	GM‐CSF inhibitor	RA	Clinical trial Phase I	190
	Namilumab	GM‐CSF inhibitor	RA	Clinical trial Phase II	383
	Gimsilumab	GM‐CSF inhibitor	COVID‐19	Clinical trial Phase II	384
	Otilimab	GM‐CSF inhibitor	COVID‐19	Clinical trial Phase II	385
	Lenzilumab	GM‐CSF inhibitor	COVID‐19	Clinical trial Phase III	386
	TJ003234	GM‐CSF inhibitor	COVID‐19	Clinical trial Phase II/III	306
	Mavrilimumab	GM‐CSFR inhibitor	RA	Clinical trial Phase II	387
			COVID‐19	Clinical trial Phase III	306
CSF‐1	Masitinib	CSF‐1R inhibitor	MS	Clinical trial Phase III	249
			ALS	Clinical trial Phase III	248
	Emactuzumab (RG7155)	CSF‐1R inhibitor	Enosynovial giant cell tumor	Clinical trial Phase III	327
	Pexidartinib (PLX3397)	CSF‐1R inhibitor	Enosynovial giant cell tumor	Marketed	329
CCR/CCL	Cenicriviroc	CCR2/CCR5 inhibitor	NAFLD	Clinical trial Phase III	271
	Leronlimab	CCR5 inhibitor	COVID‐19	Clinical trial Phase III	388
	PF‐04136309	CCR2 inhibitor	Pancreatic cancer	Clinical trial Phase II	315
	Carlumab	CCL2 inhibitor	Castration‐resistant prostate cancer	Clinical trial Phase II	316
	Maraviroc	CCR5 inhibitor	Colorectal cancer	Clinical trial Phase I	317
	Vicriviroc	CCR5 inhibitor	Colorectal Cancer	Clinical trial Phase II	317
CXCR/ CXCL	Plerixafor	CXCR4 inhibitor	Glioblastoma	Clinical trial Phase I/II	321
	Motixafortide	CXCR4 inhibitor	Pancreatic ductal adenocarcinoma	Clinical trial Phase II	389
TLR	Eritoran	TLR4 antagonist	COVID‐19	Clinical trial Phase III	390
	PUL‐042	Synthetic ligands for TLR 2/6/9	COVID‐19	Clinical trial Phase II	306
	Famotidine	Inhibit TLR3	COVID‐19	Clinical trial Phase IV	391
PPAR	Pioglitazone	PPARγ agonist	AD	Clinical trial Phase III	392
			T2DM	Marketed	393
			NAFLD	Clinical trial Phase IV	394
			COVID‐19	Clinical trial Phase IV	306
	Rosiglitazone	PPARγ agonist	AD	Clinical trial Phase III	395
			T2DM	Marketed	396
			NAFLD	Clinical trial Phase IV	394
	TZDs	PPARγ agonist	T2DM	Marketed	396
			NAFLD	Clinical trial Phase IV	394
			COVID‐19	Clinical trial Phase IV	306
	Saroglitazar	PPARα/PPARγ agonist	NAFLD	Clinical trial Phase IV	306
	Aleglitazar	PPARα/PPARγ agonist	NAFLD	Clinical trial Phase III	280
	Elafibranor	PPARα/PPARδ agonist	NAFLD	Clinical trial Phase III	281
	Lanifibranor	Pan‐PPAR agonist	NAFLD	Clinical trial Phase III	282
GLP‐1R	Exenatide	GLP‐1R agonist	T2DM	Marketed	394, 397
			NAFLD	Clinical trial Phase IV	394
	Liraglutide	GLP‐1R agonist	T2DM	Marketed	397
			NAFLD	Clinical trial Phase IV	394
	Lixisenatide	GLP‐1R agonist	T2DM	Marketed	397
	Extended‐ release exenatide	GLP‐1R agonist	T2DM	Marketed	397
	Albiglutide	GLP‐1R agonist	T2DM	Clinical trial Phase IV	397
	Dulaglutide	GLP‐1R agonist	T2DM	Marketed	397
			NAFLD	Clinical trial Phase IV	394
	Semaglutide	GLP‐1R agonist	T2DM	Marketed	397
			NAFLD	Clinical trial Phase IV	394
S1PR	Fingolimod	S1PRs (1, 3, 4, 5) agonist	MS	Marketed	398
			ALS	Clinical trial Phase II	251
			COVID‐19	Clinical trial Phase II	399
	Ozanimod	S1PRs (1, 5) agonist	COVID‐19	Marketed	400
SIRPα/CD47	Hu5F9‐G4	CD47 inhibitor	Non‐Hodgkin lymphoma, colorectal neoplasms	Clinical trial Phase I/II	335
	CC‐90002	CD47 inhibitor	Acute myeloid leukemia	Clinical trial Phase I	336
FXR	Obeticholic acid	FXR agonist	NAFLD	Marketed	274, 275
Drugs targeting signaling pathway in macrophage					
FcR pathway	Spebrutinib	BTK inhibitor	RA	Clinical trial Phase II	192
	Fenebrutinib	BTK inhibitor	RA	Clinical trial Phase III	193
	Tolebrutinib	BTK inhibitor	MS	Clinical trial Phase III	208
	Evobrutinib	BTK inhibitor	MS	Clinical trial Phase III	401
	Ibrutinib	BTK inhibitor	COVID‐19	Clinical trial Phase II	306
	Acalabrutinib	BTK inhibitor	COVID‐19	Clinical trial Phase III	306
NF‐κB pathway	NSAIDs	Inhibit NF‐κB	RA, IBD, COVID‐19	Marketed	306, 363, 402
	Corticosteroids	Inhibit NF‐κB and AP‐1	RA, IBD, MS, COVID‐19	Marketed	363, 398, 402, 403
	Cyclophosphamide	Inhibit NF‐κB	RA	Marketed	402
	Thalidomide	Inhibits the release of TNF‐α and NF‐κB activation	IBD	Clinical trial Phase III	404
	DMF	Blockade the JAK‐STAT pathway and downregulate NF‐κB	MS	Marketed	398
	COVID‐19		Clinical trial Phase IV	306
	NP001	Inhibit the NF‐κB pathway	ALS	Clinical trial Phase II	405
	Minocycline	Downregulate NF‐κB	ALS	Clinical trial Phase III	241
JAK‐STAT pathway	Tofacitinib	JAK1/2/3/TYK2 inhibitor	RA	Marketed	406
			IBD	Marketed	406
			COVID‐19	Clinical trial Phase IV	407
	Baricitinib	JAK1/2 inhibitor	RA	Marketed	406
			COVID‐19	Clinical trial Phase IV	408
	Peficitinib	JAK1/2/3 inhibitor	RA	Clinical trial Phase III	409
			IBD	Clinical trial Phase II	410
	Upadacitinib	JAK1/3 inhibitor	RA	Marketed	406
			IBD	Marketed	406
			COVID‐19	Clinical trial Phase IV	411
	Filgotinib	JAK1 inhibitor	RA	Clinical trial Phase IV	412
			IBD	Clinical trial Phase III	413
	Ritlecitinib	JAK3 inhibitor	RA	Marketed	406
			IBD	Marketed	406
	Izencitinib	Pan‐JAK inhibitor	IBD	Clinical trial Phase III	406
	Deucravacitinib	TYK2 inhibitor	IBD	Marketed	406
	Brepocitinib	JAK1/TYK2 inhibitor	IBD	Clinical trial Phase II	406
	Pacritinib	JAK2 inhibitor	COVID‐19	Clinical trial Phase II	414
	Ruxolitinib	JAK1/2 inhibitor	COVID‐19	Clinical trial Phase III	415
	Nezulcitinib	Pan‐JAK inhibitor	COVID‐19	Clinical trial Phase I	416
	Glatiramer acetate	Inhibit the JAK‐STAT pathway	MS	Marketed	398
	Laquinimod	Inhibit the JAK‐STAT, Akt and JNK pathway	MS	Marketed	398
	DMF	Blockade the JAK‐STAT pathway and downregulate NF‐κB	MS	Marketed	398
			COVID‐19	Clinical trial Phase IV	306
PI3K/Akt/ mTOR pathway	Duvelisib	Inhibit PI3Kδ and PI3Kγ	COVID‐19	Clinical trial Phase II	306
			T‐cell lymphoma	Marketed	417
	Laquinimod	Inhibit the JAK‐STAT, Akt and JNK pathway	MS	Marketed	398
	RNS60	Inhibit PI3Kγ and activate PI3Kα/β	ALS	Clinical trial Phase II	253
	Metformin	Inhibit mTOR and activate the AMPK pathway	T2DM	Marketed	265
			COVID‐19	Clinical trial Phase III	418
	Rapamycin	mTOR inhibitor	COVID‐19	Clinical trial Phase II	306
	RTB101	mTOR inhibitor	COVID‐19	Clinical trial Phase II	419
NLRP3	Colchicine	Inhibit NLRP3 activity	AS	Clinical trial Phase IV	288, 289
			COVID‐19	Clinical trial Phase IV	420
	Azithromycin	Inhibit NLRP3 activity	COVID‐19	Clinical trial Phase IV	421
	Melatonin	Inhibit NLRP3 activity	COVID‐19	Clinical trial Phase II/III	422
	Dapansutrile (OLT1177)	NLRP3 inhibitor	COVID‐19	Clinical trial Phase II	306
MAPK pathway	Losmapimod	Inhibit p38α and p38β	COVID‐19	Clinical trial Phase III	306
	Azathioprine	Inhibit the JNK phosphorylation	RA	Marketed	402
			IBD	Clinical trial Phase IV	423
			MS	Marketed	398
	Laquinimod	Inhibit the JAK‐STAT, Akt and JNK pathway	MS	Marketed	398
	18β‐ glycyrrhetinic acid	Suppress MAPK pathway	MS	Marketed	398
	Selonsertib	ASK1 inhibitor	NAFLD	Clinical trial Phase III	276
AMPK pathway	Methotrexate	Activate the AMPK pathway	RA	Marketed	402
			COVID‐19	Clinical trial Phase III	306
	Metformin	Inhibit mTOR and activate AMPK pathway	T2DM	Marketed	265
			COVID‐19	Clinical trial Phase III	418

Abbreviations: AD, Alzheimer's disease; ALS, amyotrophic lateral sclerosis; AS, atherosclerosis; BTK, Bruton's tyrosine kinase; COVID‐19, coronavirus disease 2019; CSF‐1R, colony‐stimulating factor‐1 receptor; CXCL, CXC motif ligand; CXCR, CXC chemokine receptor; DMF, dimethyl fumarate; FcR, Fc receptor; FXR, farnesoid X receptor; GLP‐1R, glucagon‐like peptide 1 receptor; GM‐CSF, granulocyte‐macrophage colony‐stimulating factor; GM‐CSFR, GM‐CSF receptor; IBD, inflammatory bowel disease; IL‐6, interleukin 6; JAK‐STAT, Janus kinase–signal transducer and activator of transcription; MABp1, monoclonal antibody against IL‐1 alpha; MAPK, mitogen‐activated protein kinase; MS, multiple sclerosis; NAFLD, nonalcoholic fatty liver disease; NF‐κB, nuclear factor kappa‐B; NSAIDs, nonsteroidal antiinflammatory drugs; PI3K, phosphatidylinositol 3‐kinase; PD, Parkinson's disease; RA, rheumatoid arthritis; S1PRs, sphingosine 1‐phosphate receptor; T2DM, type 2 diabetes mellitus; TB, tuberculosis; TLR, Toll‐like receptor; TNF‐α, tumor necrosis factor α; TZDs, thiazolidinedione.

### 4.1 Macrophages in autoimmune diseases

Autoimmune diseases refer to tissue trauma caused by excessive and persistent inflammation owing to autoantibodies or self‐reactive lymphocytes when immune tolerance is broken.^180^ In particular, macrophages have received increasing attention in the occurrence and progression of autoimmune diseases. Here, we focus on the role of macrophages in the pathogenesis of rheumatoid arthritis (RA), inflammatory bowel disease (IBD), and multiple sclerosis (MS) and summarize macrophage‐targeted therapeutics currently available.

#### 4.1.1 RA

RA is characterized by the massive infiltration of inflammatory cells and proinflammatory mediators in the synovial membrane of multiple joints. Notably, macrophages are one of the most numerous cells in the RA synovium, the number of which is positively correlated with the severity of clinical signs.^181^ The altered microenvironment in RA affects macrophage survival and proliferation and promotes M1 macrophage polarization through membrane receptor‐mediated signal transduction, including the NF‐κB, JAK‐STAT1, IRF5,^182^, ^183^ and Notch^184^ signaling pathways, thus promoting joint inflammation. In RA patients, macrophages have high mitochondrial activity and produce high levels of ATP and ROS,^185^ giving rise to oxidative damage to bone and cartilage. In addition, macrophages display enhanced resistance to apoptosis owing to sustained activation of the NF‐κB, STAT3, and PI3K signaling pathways, possibly related to the pathogenesis of RA.^186^

In view of the critical role of macrophages in the pathogenesis of RA, efforts are being made to develop macrophage‐targeted therapeutics. Several types of agents have succeeded in clinical trials for the treatment of RA. Inflammatory cytokine inhibitors targeting TNF‐α, IL‐6, IL‐1, and IL‐12/23 have been developed, among which the most widely used is anti‐TNF agents. These agents promote macrophage polarization toward the M2 phenotype and increase macrophage apoptosis, leading to the resolution of inflammation in RA.^187^, ^188^, ^189^ GM‐CSF inhibitors can decrease M1 polarization and relieve inflammation. Abundant clinical trials have affirmed the safety and efficacy of GM‐CSF or GM‐CSFR inhibitors in RA patients.^190^ In addition, five JAK inhibitors have been approved for the clinical treatment of RA and have shown comparable efficacy to TNF inhibitors.^191^ Bruton's tyrosine kinase (BTK), a component of Fc receptor (FcR) signaling pathway, is involved in the secretion of NO, TNF‐α, and IL‐1β by macrophages and is a therapeutic target for RA.^192^ An irreversible BTK inhibitor spebrutinib and a highly selective noncovalent BTK inhibitor fenebrutinib have been evaluated in phase II trials and demonstrated efficacy and safety in RA.^193^

#### 4.1.2 IBD

IBD is a chronic inflammatory disease in the gastrointestinal tract, primarily encompassing Crohn's disease and ulcerative colitis. Patients with IBD have incomplete immune tolerance and thus initiate innate and adaptive immunity in the intestine with the participation of gut microbiota, leading to inflammation that is hyperactive and difficult to self‐limit. Intestinal macrophages play a major role in the establishment and maintenance of gut homeostasis, and dysfunction of intestinal macrophages is proposed to be the basis of chronic inflammation in IBD.^194^ As with other inflammatory diseases, intestinal macrophages convert toward the M1 phenotype with the guidance of a variety of environmental signals in IBD, contributing to the overproduction of proinflammatory factors, suppression of anti‐inflammatory cytokines, and delayed clearance of bacteria.^195^, ^196^

Some of the anti‐TNF agents and anti‐IL‐12/23 agents used in RA are also widely used in IBD, and JAK inhibitors have entered late‐stage clinical trials. The well‐known mechanism for the effect of TNF‐α blockers on IBD is the induced differentiation of macrophages to the M2 phenotype and the secretion of the anti‐inflammatory cytokine IL‐10, which depends on FcR signaling.^197^, ^198^ In addition to suppressing the proinflammatory effect of several cytokines, JAK inhibitors downregulate the M1 pathway and permit macrophages to shift their balance toward a regulatory phenotype in patients with IBD.^199^

In addition to the above agents, some of the first‐line treatments for IBD can have an impact on signaling pathways to alter the proinflammatory profiles of macrophages. For instance, corticosteroids direct monocytes to differentiate toward M2 macrophages by inactivating NF‐κB and AP‐1.^200^, ^201^ Salicylate abrogates NF‐κB activation predominantly in macrophages, leading to decreased proinflammatory gene expression.^202^ The commonly used immunosuppressive drug azathioprine can exert an anti‐inflammatory effect in a mouse macrophage model by inhibiting Rac1 activity, JNK phosphorylation, and *iNOS* expression.^203^

#### 4.1.3 MS

MS is an idiopathic inflammatory demyelinating disease of the CNS that is caused by immune responses to self‐antigens. The role of microglia/macrophages in MS pathogenesis is similar to that of other autoimmune diseases mentioned above, such as oxidative damage and proinflammatory cytokine and chemokine production, resulting in inflammation and destruction in the brain.

Drugs targeting chemokines, especially the CCL2/CCR2 axis, achieve satisfactory efficacy in preclinical studies and animal models; however, they encounter challenges in clinical research.^204^ AZD1480, a JAK1/2 inhibitor, deactivates the M1 phenotype and diminishes disease severity in multiple preclinical models of MS,^205^ implying the potential possibility of applying JAK‐STAT inhibitors in the clinic. Furthermore, the GM‐CSF antibody MOR103 and the BTK inhibitors tolebrutinib and evobrutinib for the treatment of MS have entered the clinical trial stage.^206^, ^207^, ^208^

Fingolimod (FTY720) is an agonist of the S1P receptor, which skews microglial polarization to the M2 phenotype, decreases the expression of inflammatory cytokines and induces apoptosis by modulating several signaling pathways involving STAT1/3, IRF8, p38, PI3K/Akt, and NF‐κB.^209^, ^210^, ^211^ Glatiramer acetate, laquinimod, and dimethyl fumarate (DMF) can block the JAK‐STAT pathway and have beneficial effects.^212^ In addition, laquinimod and DMF can reduce the M1 phenotype of microglia and the secretion of proinflammatory mediators by suppressing the expression of *NF‐κB* and reducing the activity of the JNK and Akt pathways, respectively.^213^, ^214^ 18β‐glycyrrhetinic acid significantly decreases the proinflammatory profiles of microglia by suppressing the MAPK signaling pathway and promotes remyelination in an experimental autoimmune encephalomyelitis model.^215^

### 4.2 Macrophages in neurodegenerative diseases

Neurodegenerative diseases feature progressive and irreversible loss of selective populations of neurons, mainly AD, PD, and ALS, according to primary clinical features.^216^ As the role of microglia/macrophages in disease pathogenesis and progression is gradually being recognized, microglia have the potential to be prime targets for future therapeutic strategies.

#### 4.2.1 Alzheimer's disease (AD)

AD is the most prevalent degenerative disease of the CNS and is characterized by progressive memory loss, behavioral change, and cognitive dysfunction. One of the most characteristic pathological features of AD is the deposition of Aβ and consequently the formation of neurotic plaques, which is considered by many scholars to be the initial factor of neuronal degeneration.^217^ Other pathological features of AD are tau protein hyperphosphorylation and abnormal aggregation, leading to the formation of neurofibrillary tangle and the dysfunction of normal neurons and synapses. Notably, activated microglia elevate tau hyperphosphorylation in a manner dependent on CX3CR1 and IL‐1/p38 MAPK^218^ and drive tau propagation and toxicity via NF‐κB signaling.^219^ The intricate interactions between the two features of AD and neuroinflammation primarily triggered by microglia have become a hot topic in this field of research.

TNF is a common target to suppress inflammation in various diseases, and the same is true in AD. However, whether to use TNF‐α inhibitors for the treatment of AD is a matter of debate both in clinical trials and preclinical research. Pilot studies reported improvement in efficacy variables with treatment with etanercept, a TNF‐alpha inhibitor in patients with AD,^220^, ^221^ while a double‐blind phase II study observed no statistically significant changes in cognition, behavior, or global function.^222^ Meanwhile, although there is evidence that anti‐TNF‐α reduces the levels of amyloid plaques and tau phosphorylation,^223^ a study argues against long‐term use of pan‐anti‐TNF‐α inhibitors in AD, owing to the importance of intact TNF‐α receptor signaling for microglial‐mediated uptake of extracellular Aβ peptide pools.^224^ Recently, a novel strategy of targeting the TNF pathway that selectively inhibits proinflammatory and neurotoxic pathways mediated by TNFR1 while preserving the neuroprotective pathway mediated by TNFR2 to counteract Aβ pathology has been proposed,^225^ which may advance immunotherapy for AD.

Nuclear receptor PPARγ is another promising target. Activated PPARγ in microglia can stimulate NF‐κB (and, to a lesser extent, AP‐1 and STATs) to reduce the production of inflammatory mediators.^226^ The results of clinical trials for PPARγ agonists in AD are positive overall.^227^, ^228^ Notably, in some trials, the efficacy of PPARγ agonists in individuals depends on their ApoE genotype.^229^ The basis of this effect remains unexplained.

Moreover, attempts have been made to modulate other targets in microglia, such as p38, TLRs, NF‐κB, NLRP3, TREM‐2, and the CD200‐CD200R axis,^230^, ^231^, ^232^, ^233^, ^234^ which need to be further investigated and evaluated in clinical trials before being moved from bench to bedside.

#### 4.2.2 Parkinson's disease (PD)

PD is a class of movement disorders characterized by bradykinesia, muscular rigidity, rest tremor, and postural and gait impairment. Its key pathological features include loss of dopaminergic neurons within the substantia nigra of the midbrain, Lewy pathology with α‐synuclein aggregation, and neuroinflammation.^235^ As one of the key cellular players in neuroinflammation, microglia bridge the three pathological features during the pathogenesis of PD.

The induction effect of α‐synuclein on neuroinflammation depends in part on the activation of the TLR/NF‐κB pathway in activated microglia, which causes the increased transcription of *NLRP3*, *pro‐IL‐1β*, and *pro‐IL‐18*. In α‐synuclein high expression mouse models, inhibiting the component of the TLR‐NF‐κB‐NLRP3 axis demonstrated a reduction in neuroinflammation, nigral dopaminergic degeneration and loss, and behavioral deficits.^236^ Although not yet in clinical trials, many PPAR agonists have been recognized to protect nerve cells from inflammation in preclinical studies of PD. The administration of the PPARγ agonist pioglitazone in in vitro experiments or animals experiencing PD offered a neuroprotective effect with a decrease in NF‐κB and microglial activation and the preservation of dopamine striatal fibers and substantia nigra nerve cells. PPARβ/δ, PPARα, and PPARα/γ agonists can exert similar actions.^237^ CX3CL1 is a unique chemokine produced in neurons and signals through its sole receptor CX3CR1 expressed on microglia. Studies have shown that CX3CL1/CX3CR1 signaling is closely related to the disease progression of PD. Increased CX3CL1 signaling decreases neuronal loss from α‐synuclein exposure by modulating microglia to a more neuroprotective and less proinflammatory phenotype, and loss of CX3CR1 on microglia leads to an increase in PD pathology. Administration of CX3CL1 via a virus or osmotic minipumps in animal models of PD can have an effect of reducing neurodegeneration and microglia activation, holding great promise.^238^

#### 4.2.3 Amyotrophic lateral sclerosis (ALS)

ALS is a motor neuron disease that is characterized by the progressive loss of both upper and lower motor neurons. It can affect any voluntary muscle, resulting in heterogeneous presentations of motor deficits. The degree of microglial activation in the motor cortex is significantly correlated with the severity of clinical signs in ALS.^239^

Minocycline, a widely used broad‐spectrum antibiotic, selectively inhibits microglia by downregulating NF‐κB, that is, it suppresses M1 microglia during the progressive phase but does not affect the function of M2 microglia.^240^ Nevertheless, in a phase III randomized trial, minocycline was observed to have even a harmful effect on patients with ALS after continuous administration.^241^ In addition, minocycline does not confer long‐term benefit in patients with other neurodegenerative diseases,^242^, ^243^ indicating that the application of minocycline in neurodegenerative diseases requires additional caution.

Novel treatment approaches are under development for patients with ALS. NP001, a formulation of purified sodium chlorite, converts into taurine chloramine within monocytes/macrophages that induce proinflammatory macrophages to transform to a phagocytic state by inhibiting the NF‐κB pathway.^244^ Its safety and early efficacy in ALS have been affirmed by phase I and II clinical trials.^245^, ^246^ Masitinib, an oral tyrosine kinase inhibitor (TKI), prevents microglial proliferation, migration, and inflammatory mediator production via selectively inhibiting CSF‐1R in the SOD1 mutant rat model of ALS.^247^ A phase II trial showed that masitinib can benefit ALS patients,^248^ as well as MS patients.^249^ Clinical trials for other common targets in macrophage signaling pathways, such as TNF‐α, IL‐6, IL‐1, PI3K/Akt, and sphingosine 1‐phosphate receptor (S1PR), are being conducted in ALS patients, among which the S1PR agonist fingolimod, IL‐6R antagonist tocilizumab, and IL‐1R antagonist anakinra demonstrate good safety and tolerability,^250^, ^251^, ^252^ and a modulator of the PI3K/Akt pathway RNS60 shows a positive effect,^253^ whereas the trial of the TNF‐α antagonist thalidomide has failed.^254^

### 4.3 Macrophages in metabolic diseases

Metabolic homeostasis in the human body depends on the coregulation of metabolic organs such as the pancreas, liver, and adipose tissue. Meanwhile, the immune response and metabolic regulation are highly integrated, and their proper function depends on each other.^3^ Once this homeostasis is broken, it will trigger a series of chronic metabolic diseases, particularly obesity, type 2 diabetes mellitus (T2DM), nonalcoholic fatty liver disease (NAFLD), and cardiovascular diseases.^255^

#### 4.3.1 T2DM

Obesity is the major cause of insulin resistance. However, obese patients do not always develop T2DM. Hyperglycemia occurs only when islet inflammation impairs the function of β cells, preventing them from producing enough insulin to compensate for insulin resistance. Macrophages that present the proinflammatory classically activated M1‐like phenotype dominate in T2DM islet inflammation,^256^ and the ratio of M1/M2 is related to the degree of insulin resistance.^257^ Circulating factors such as glucose and free fatty acids (FFAs) induce macrophage secretion of cytokines, particularly IL‐1β and TNF‐α, by binding to TLR4 on macrophages and activating the NF‐κB pathway.^258^ These cytokines promote β cell apoptosis and attenuate glucose‐stimulated insulin secretion by activating the NF‐κB and JNK pathways and deteriorating ER stress in β cells.^258^

Considering the injurious effect of TNF‐α and IL‐1 produced by macrophages on the insulin signaling pathway, insulin resistance and glycemic control can theoretically be improved by therapies targeting these cytokines. In fact, the treatment of neutralizing cytokines is indeed efficacious in diabetic rodent models.^259^, ^260^ However, clinical trials have been less cheerful with mixed results.^261^, ^262^, ^263^, ^264^

PPARγ agonists, metformin, and glucagon‐like peptide‐1 receptor agonists (GLP‐1RAs) are three widely used and effective classes of antidiabetic agents. Their inhibitory effect on macrophages likely helps to explain their glucose‐lowering role. PPARγ agonists used in T2DM include thiazolidinedione, pioglitazone, and rosiglitazone, the modulation mechanism of which has been discussed in the AD section. Metformin has anti‐inflammatory actions by suppressing the activity of the NLRP3 inflammasome and the maturation of IL‐1β in macrophages by activating the AMPK pathway.^265^ GLP‐1 is a kind of brain‐gut peptide secreted by ileal endocrine cells. Various types of cells, including macrophages, express the GLP‐1 receptor (GLP‐1R). After treatment with GLP‐1RAs or GLP‐1 in vivo and in vitro, the phenotype of macrophages is modified toward M2, and inflammatory cytokine secretion is inhibited, possibly because the STAT pathways are affected.^266^ Enthusiasm for the development of GLP‐1RAs is growing rapidly, and there are already seven clinically approved GLP‐1R agonists currently used for T2DM.

#### 4.3.2 NAFLD

FFAs released from adipocytes, damaged hepatocytes, endotoxins, and translocated bacteria resulting from increased intestinal permeability and changes in gut microbiota are factors that activate Kupffer cells in NAFLD.^267^ Activated macrophages promote the development of hepatic steatosis, steatohepatitis, fibrosis, and ultimately hepatic failure through inflammatory cytokines, oxidative stress, and accumulated endotoxins owing to impaired phagocytic activity.

At present, there are a variety of macrophage signaling‐targeted therapeutic approaches for NAFLD treatment that have shown efficacy in disease models.^268^ Here, we discuss only those therapeutic approaches that have already been evaluated in human trials owing to space limitations.

Cenicriviroc, a dual CCR2/CCR5 inhibitor, can inhibit monocyte‐derived macrophage recruitment mediated by CCL2 and significantly relieve hepatic steatosis and liver fibrosis in experimental models.^269^ Phase II trials substantiated that cenicriviroc was well tolerated and exhibited an antifibrogenic effect in patients with nonalcoholic steatohepatitis^270^, ^271^; however, a phase III trial was recently terminated owing to undesirable results in the interim analysis.^272^ Farnesoid X receptor (FXR) is another promising nuclear receptor target. FXR signaling not only is central to metabolic homeostasis but also reduces liver inflammation by skewing macrophages toward the M2 phenotype and downregulating inflammatory cytokines.^273^ Its agonist, obeticholic acid, has obtained satisfactory results in patients with NAFLD.^274^, ^275^ ASK1, a serine/threonine kinase, promotes inflammation and fibrosis partly by activating MAPK pathways in macrophages. A phase II clinical trial demonstrated the efficacy of the ASK1 inhibitor selonsertib in relieving liver fibrosis in NAFLD patients.^276^ The two aforementioned classes of agents, GLP1RAs and PPAR agonists, have also been extensively evaluated in NAFLD patients.^277^, ^278^ Moreover, PPARα, PPARδ, and PPARγ have similar effects on macrophages. Several drugs have been developed to target multiple receptor subtypes rather than one, including the PPARα/PPARγ agonists saroglitazar and aleglitazar, the PPARα/PPARδ agonist elafibranor, and the pan‐PPAR agonist lanifibranor. All of them are being investigated in patients with NAFLD in clinical trials.^279^, ^280^, ^281^, ^282^

#### 4.3.3 Atherosclerosis (AS)

AS is the pathological basis of many cardiovascular diseases, such as myocardial infarction (MI), stroke, and cardiovascular death. Macrophages are considered to be the central cellular effectors of AS progression and run throughout all stages of disease development.^283^ The polarization of macrophages largely affects the formation and stability of atherosclerotic plaques. M1 macrophages are found to be higher in unstable plaques or near the lipid core within advanced plaques and dominate the infarction and rupture of plaques, whereas M2 macrophages are the opposite. Pro‐inflammatory stimuli existing in the plaque microenvironment induce M1 polarization, breaking the balance between M1 and M2.

Although measures to prevent AS have mainly focused on lipid‐lowering treatment until now, progress has been made in targeting inflammatory pathways. The well‐known CANTOS trial is the first large‐scale, randomized, double‐blind trial in human AS. This trial tested the efficacy of canakinumab, an IL‐1β monoclonal antibody, in 10,061 patients with previous MI on the basis of standard therapy.^284^, ^285^ This trial confirmed that canakinumab could further reduce the recurrence and total number of adverse cardiovascular events in patients with prior MI independent of lipid‐level lowering, providing direct evidence for the inflammation hypothesis of AS and a basis for the subsequent development of more inflammation‐targeted drugs. Beyond IL‐1β, the application of agents targeting other cytokines, such as TNF, IL‐6, and IFN‐γ, which are related to macrophage signaling, is also being explored in AS, although the results may not be as inspiring as those of IL‐1β.^286^

Colchicine, which is used for gouty arthritis and familial Mediterranean fever, has been extended to AS. The mechanism of action of colchicine is to disrupt tubulin and subsequently downregulate multiple inflammatory pathways and modulate innate immunity. Its impact on macrophages is mainly reflected in suppressing the release of various proinflammatory substances, such as ROS, NO, and TNF‐α, and inhibiting the maturation of IL‐1β via the NALP3/caspase‐1 or ROCK/caspase‐1 pathway.^287^ For the application of colchicine in AS, there are two large clinical trials, COLCOT and LoDoCo2, and both show a significantly reduced risk of cardiovascular events for patients with a recent MI who received colchicine.^288^, ^289^ According to the long‐term follow‐up of LoDoCo2, the benefit of colchicine continues to accumulate over time.^290^

### 4.4 Macrophages in infectious diseases

When host defense is challenged by invading infectious microbes, macrophages are recruited and differentiate into a proinflammatory phenotype after engagement and recognition of pathogens. These macrophages are activated through inflammatory signaling pathways and can secrete inflammatory mediators to promote pathogen killing in the early stages of the disease; however, they can cause tissue damage and even organ failure in diseases such as tuberculosis (TB) and coronavirus disease 2019 (COVID‐19).

#### 4.4.1 TB

TB, a worldwide infectious disease that has led to major health issues, is caused by *Mycobacterium tuberculosis* (*Mtb*), an intracellular pathogenic bacterium.^291^ The innate immune system is first challenged by *Mtb* infection, in which macrophages can present dual characteristics of eliminating pathogenic *Mtb* and establishing niches for the persistence and dissemination of *Mtb*.^291^, ^292^

Currently, there are two approaches targeting macrophages in the treatment of TB.^293^ One is to amplify cellular antimicrobial mechanisms, thereby enhancing the effectiveness of anti‐*Mtb* chemotherapy. Imatinib, a TKI, can promote acidification and maturation of macrophage phagosomes, thus reducing intracellular *Mtb* survival.^294^, ^295^ Gefitinib, an inhibitor of EGFR, induces autophagy, thus restricting *Mtb* growth and replication in macrophage cytoplasm.^296^ The other approach is to reduce inflammation caused by macrophages, such as the administration of TNF blockers, JAK inhibitors, thalidomide analogs, statins, and PPARγ agonists in severe *Mtb* infections.^293^, ^297^, ^298^, ^299^, ^300^ However, these immunomodulators might reactivate latent *Mtb* infection or exacerbate active *Mtb* infection if not administered concurrently with antimycobacterial chemotherapy. Therefore, further research on drug dose and clinical value is needed.

#### 4.4.2 COVID‐19

COVID‐19 is a contagious viral disease caused by SARS‐CoV‐2 and has become a global health concern due to its worldwide pandemic. Innate immunity functions as the first line of host defense, in which macrophages play a crucial role.^301^ While the undeniable antiviral effect of macrophages is widely accepted, hyperinflammation and the subsequent cytokine storm triggered by overactivated macrophages are gaining concern as complications observed in severe COVID‐19 patients.^302^

Macrophages are first activated through the TLR4/TLR7‐TRAF6‐NF‐κB pathway by identifying the SARS‐CoV‐2 structure. Meanwhile, the virus enters the macrophage cytoplasm through the ACE2 receptor, activating the NLRP3 inflammasome in macrophages. Chemokines released by infected epithelial cells and cytokines released by activated NK cells and T cells further promote the recruitment and activation of macrophages through the JAK‐STAT pathway.^302^, ^303^ In mild or moderate COVID‐19 patients, activated macrophages defend against SARS‐CoV‐2 infection by inflammatory cytokine production and suicide by pyroptosis. However, in severe cases, a reduced or delayed type I IFN response leads to increased cytopathic effects and enhanced chemokine release, thus contributing to sustained recruitment of macrophages. The continuous activation of macrophages leads to the overproduction of inflammatory cytokines and cytokine storms that eventually cause multiple organ failure and even death in patients.^301^, ^302^, ^304^

It has been shown that blocking the entry and replication of virus in macrophages, as well as inhibiting the production of inflammatory factors, inflammasome activation and the type I IFN response can reduce the overactive immune inflammatory response. On the one hand, antiviral drugs remdesivir, molnupiravir, and nirmatrelvir‐ritonavir are proven to be effective in preventing hospitalization and death.^305^ On the other hand, medications attempting to block inflammatory pathways, such as TLR/NF‐κB, NLRP3, and JAK‐STAT or inflammatory cytokines, including IL‐1β, IFNs and TNF‐α, are currently under clinical trials.^302^, ^306^ The results of clinical trials that have already been published show that inflammatory cytokine or signaling pathway inhibition therapies indeed improve the clinical outcomes of most patients, principally reflected in more rapid clinical symptom improvement, reduced inflammatory indices, decreased oxygen and assisted ventilation requirements, and a lower risk of mortality.

### 4.5 Macrophages in cancer

The approach for cancer treatment in recent years has focused on the tumor microenvironment (TME), which includes malignant, endothelial, stromal, and immune cells. Tumor‐associated macrophages (TAMs) are a plastic and heterogeneous cell population that account for the largest fraction of the myeloid infiltrate in most of human solid malignancies.^48^ TAMs can arise from both TRMs and circulating monocytes.^307^ And TAMs tend to be considered M2‐like macrophages as they have much of the representative properties of M2 macrophages. However, recently developed techniques such as single‐cell RNA sequencing have shown that TAMs rarely display bona fide M1 or M2 phenotypes, and the complexity of these cells cannot easily be resolved with the traditional binary “M1‐M2” system.^5^, ^308^, ^309^ In respect to the function of TAMs, it is generally agreed that M1‐like TAMs exert antitumor effects,^310^ while M2‐like TAMs promote the occurrence, metastasis, and angiogenesis of tumor cells, and inhibit the anti‐tumor immune response mediated by T cells.^311^ The two phenotypes of TAMs can be converted into each other in response to environmental factors.^312^ Therefore, targeting macrophage‐related signaling pathways to promote TAM depletion, inhibit monocyte recruitment, enhance TAM phagocytosis, and reprogram M2‐like TAMs to M1‐like TAMs could be promising therapeutic strategies for enhancing anti‐cancer immunity.

#### 4.5.1 Targeting TAM recruitment

In tumor‐bearing mice, blockade of CCL2/CCR2 signaling inhibits the recruitment of inflammatory monocytes, delays cancer growth and metastasis, reduces postsurgical recurrence, and enhances survival.^313^ However, interruption of CCL2 inhibition leads to an unexpected influx of monocytes from the bone marrow, which enhances cancer cell metastases and accelerates death in mouse models of metastatic breast cancer.^314^ Further studies are needed to avoid this lethal rebound.

Several clinical trials targeting CCR2/CCL2 are ongoing. For example, a phase 1b trial in patients with borderline resectable and locally advanced pancreatic cancer showed decreased infiltration of TAMs in tumors and increased responsiveness to chemotherapy in patients treated with the small‐molecule CCR2 inhibitor PF‐04136309 combined with folinic acid, fluorouracil, irinotecan, and oxaliplatin (FOLFIRINOX), compared with those receiving FOLFIRINOX alone.^315^ However, another phase 2 study of a human monoclonal antibody against CCL2, carlumab (CNTO 888), showed no antitumor activity as a single agent in patients with metastatic castration‐resistant prostate cancer.^316^

In mouse models of various cancer types, including colorectal cancer (CRC), gastric cancer, and breast cancer, CCR5 inhibitors, including maraviroc, vicriviroc, and TAK‐779, have demonstrated antitumor effects.^317^ In PDXs of malignant phyllodes tumors, CCR5 blockade with maraviroc showed macrophage repolarization with antitumor effects.^318^ These antitumor effects were then confirmed in several clinical trials. A phase I trial with the CCR5 antagonist maraviroc in patients with liver metastases of advanced refractory CRC showed mitigation of the tumor‐promoting microenvironment and objective tumor responses (NCT01736813).^319^ The combination of maravironic and pembrolizumab also showed beneficial activity and toxicity in mismatch repair‐proficient CRC (NCT03274804).^320^

Preclinical studies with intracranial gliomas in both mice and rats clearly demonstrate that the marked increase in macrophage entry into irradiated tumors is mediated by the CXC motif ligand (CXCL) 12‐CXC chemokine receptor (CXCR) 4 pathway and that inhibition of this pathway blocks macrophage entry, thereby improving local control and survival after radiation therapy.^321^ A phase I/II study in patients with newly diagnosed glioblastoma using the reversible CXCR4 inhibitor plerixafor after radiation was conducted, and the results showed that the infusion of plerixafor was well tolerated as an adjunct to standard chemoirradiation and that the local control of tumor recurrence was improved.^321^

#### 4.5.2 Targeting TAM activation and proliferation

Several small molecules and antibodies of the CSF‐1/CSF‐1R axis have been developed and tested in a substantial array of animal models. For example, in one study, RG7155, a monoclonal antibody that inhibits CSF‐1R activation, caused macrophage cell death in vitro and reduced TAM densities accompanied by an increase in the CD8(+)/CD4(+) T‐cell ratio in vivo.^322^ Furthermore, experimental studies have started to assess combinations of CSF‐1R inhibitors with chemotherapy,^323^ radiotherapy,^324^ or ICB.^307^ In mouse models of different cancer types, such as glioblastoma,^324^ prostate cancer,^325^ and pancreatic tumors,^326^ CSF‐1R has been observed to reduce side effects and potentiate the efficacy of radiotherapy.^324^, ^325^, ^326^

In addition, various drugs targeting CSF‐1(R), including small‐molecule inhibitors such as sotuletinib, ARRY‐382, and pexidartinib and antagonistic monoclonal antibodies such as emactuzumab, cabiralizumab, LY3022855, and axatilimab, have been or are being tested in clinical trials. For patients with tenosynovial giant cell tumor, a rare, locally aggressive neoplasm that overexpresses CSF‐1, objective and complete responses were observed during a phase 1 study of emactuzumab,^327^ a phase 2 trial of PLX3397,^328^ and a randomized phase 3 trial of pexidartinib.^329^ Additionally, the effectiveness of the combination therapy in animal trials led to a number of clinical trials combining CSF‐1 or CSF‐1R inhibitors with radiotherapy, ICBs, or chemotherapeutic agents. For example, a phase 1 study of emactuzumab as a single agent or in combination with paclitaxel in patients with advanced solid tumors revealed depletion of immunosuppressive M2‐like macrophages (NCT01494688).^330^

#### 4.5.3 Strengthening TAM phagocytosis

Tumor cells overexpress *CD47* in many cancer types, disguising them as healthy cells and avoiding phagocytosis. Therefore, strategies targeting the CD47/SIRPα axis to enable tumor cell killing through cellular phagocytosis have emerged as promising cancer immunotherapies.^331^

In a variety of immunocompetent mouse tumor models, including ovarian cancer,^332^ breast cancer,^333^ and pancreatic cancer,^334^ treatment with anti‐CD47 antibodies stimulated macrophage phagocytosis in vitro and suppressed tumor growth in vivo.

Clinical trials are underway for both solid and hematological malignancies using anti‐CD47 antibodies. To date, anti‐CD47 antibodies such as Hu5F9‐G4,^335^ CC‐90002,^336^ and SRF231^337^ are under phase 1 clinical trials. Owing to the expression of CD47 on platelets and red blood cells, potential on‐target toxicity, in particular anemia and thrombocytopenia, is seriously considered. As an approach to achieving greater specificity toward tumors, bispecific agents have been generated. For example, a novel affinity‐tuned bispecific antibody targeting CD47 and PD‐L1 to antagonize both innate and adaptive immune checkpoint pathways is under clinical trial (NCT04881045).^338^

As mentioned above, Siglec‐10 is an inhibitory I‐type lectin that can be expressed by TAMs and binds the potent anti‐phagocytic signal CD24 that is overexpressed in several solid tumor types. The binding of CD24 to Siglec10 elicits inhibitory signaling that blocks the cytoskeletal rearrangement required for cellular engulfment by macrophages. Clinical data have shown that CD24 expression is significantly higher in triple‐negative breast cancer cells than in normal breast cells.^339^ Moreover, stratification of patients by CD24 expression revealed an increased relapse‐free survival for patients with ovarian cancer and an overall survival advantage for patients with breast cancer with lower CD24 expression, and anti‐SIGLEC10 rescued the macrophage capability to limit tumor growth.^340^

#### 4.5.4 Targeting M2‐M1 repolarization

The NF‐κB signaling pathway is important in cancer‐related inflammation and malignant progression. Studies have shown that when NF‐κB signaling is inhibited specifically in macrophages in the TME, they switch to M1 and become cytotoxic to tumor cells.^341^, ^342^ Inhibition of the NF‐κB pathway in liver macrophages by genetic deletion of IKKβ results in a marked reduction in tumor onset and load.^343^ A micellar nanodrug effectively functions in M2‐to‐M1 repolarization via M2‐targeted codelivery of IKKβ siRNA and the STAT6 inhibitor AS1517499, which suppresses tumor growth and metastasis. Because the M2‐targeting peptides are hidden in the pH‐sheddable polyethylene glycol (PEG) corona so that active targeting of M2‐like macrophages is triggered only in the acidic TME rather than the neutral‐pH environment in healthy organs, immune side effects are reduced.^344^

PI3K, the upstream regulator of Akt, has been shown to mediate M2 macrophage phenotypes. Activation of PI3Kγ signaling in macrophages has been reported to drive TAM immunosuppressive activities in models of lung cancer,^345^ pancreatic cancer,^346^ and melanoma.^347^ The pharmacologic inhibition of PI3Kγ has shown the ability to reprogram TAMs and increase T‐cell recruitment into tumors, resulting in tumor growth inhibition. Accordingly, several PI3K inhibitors are being tested for different cancer indications, such as duvelisib (dual PI3Kδ/γ inhibitor) in a phase 1 trial and preclinical models of T‐cell lymphoma,^348^ alpelisib (α‐specific PI3K inhibitor) for patients with epithelial ovarian cancer,^349^ and umbralisib (dual PI3Kδ/CK1ε Inhibitor) in patients with relapsed or refractory indolent lymphoma.^350^

## 5 DISCUSSION

Macrophages are widely present in almost all tissues of adult and play important roles in physiological and pathological processes. Although extraordinary efforts have been made to understand the functions and regulating mechanisms of macrophages, our evolving knowledge about the intricate signaling network within and between macrophage and its microenvironment is still not satisfactory yet.

The crosstalk of different signaling pathways and the bidirectional effect of the same signaling pathway leads to the complexity of macrophage regulation. First, the perception and integration of various external stimuli by macrophages lead to the activation of multiple signaling pathways and significant crosstalk of different signaling pathways, while the complicated crosstalk of signaling pathways can cause synergistic or antagonistic effects in macrophage regulation. An example of the synergistic regulation of macrophage inflammatory responses is the crosstalk between PI3K/Akt/mTOR and TLRs/NF‐κB signaling to promote inflammatory responses in *Streptococcus uberis* infection.^351^ In contrast, an example of the antagonism of different signaling pathways in macrophages to maintain macrophage immune homeostasis is that immunosuppressive receptors antagonize the signal transduction mediated by activatory receptors such as FcR, through ITIMs, to inhibit macrophage phagocytosis and cytokine production.^156^ Second, the same signaling pathway may have a bidirectional regulatory effect on macrophages, which means that the signaling pathway not only participates in the M1 macrophage polarization but also can promote the M2 polarization of macrophages under certain conditions. This bidirectional regulatory effect of the same signaling pathway on macrophages depends on factors such as different ligand–receptor interactions, intensity and duration of stimulation, phosphorylation sites, and kinase isoforms. For instance, LPS upregulates *CCL2* expression through TLR4/MyD88 signaling, which activates JNK and promotes M1 polarization;^352^ however, in IL‐4‐activated macrophages, scavenger receptor 1 leads to JNK activation through K63 ubiquitination, which promotes the polarization of macrophages to M2.^353^ In this regard, JNK signaling pathway plays a bidirectional role in macrophage polarization.

It is the complexity of the associated regulatory signaling pathways that allows macrophages to exhibit a remarkable degree of functional and phenotypic plasticity and heterogeneity in response to environmental stimuli. Many of these macrophage phenotypes are tissue‐ and environment‐specific and play different or even opposing roles in human diseases. We have outlined various types of antibody‐based drugs and small molecule drugs targeting environmental signals or macrophage receptors and intracellular signaling pathways. These drugs exert therapeutic effects by blocking or activating macrophage‐related signaling pathways and gene transcription to alter the functional or physiological properties of macrophages.

However, with the development of drugs aiming at different molecular targets of macrophages, the next challenge is to effectively and selectively deliver these drugs to specific disease‐related macrophage subpopulations to improve efficacy and reduce off‐target effects. Studies have found that targeting of macrophages by phagocytosis of nanoparticles or liposomes or by antibodies binding to macrophage‐specific receptors can improve the therapeutic efficacy of drugs.^354^, ^355^ For example, a mannose‐modified nanoparticle is masked by acid‐sensitive PEG to prevente recognition by TRMs in a neutral pH. While in acidic TME, PEG is shed thereby exposing mannose and binding to mannose receptors on TAMs to achieve intratumor TAM targeting.^356^ Besides, lactoferrin‐modified liposomes (LF‐lipo) can specifically bind to low‐density lipoprotein receptor‐related protein expressed on activated colonic macrophages for cell‐targeted anti‐inflammatory therapy, showing enhanced therapeutic efficacy in a mouse colitis model.^357^ Moreover, bispecific monoclonal antibodies, which may overcome off‐target effect by the increased infinity with disease‐specific targets, have dual localization and therapeutic roles and are able to precisely target macrophages in the specific site. A classic example in cancer is bispecific antibodies targeting CD47 and tumor‐associated antigens such as CD20^358^ and PD‐L1^359^ that specifically target macrophages in tumor site, promoting phagocytosis of tumor cells by macrophages but sparing the host cells that do not express the tumor antigens such as platelets and red blood cells, showing limited toxicity.

Furthermore, due to the complexity of macrophage regulatory pathways, the efficacy of a single drug is limited, and combination therapy is a promising direction. For instance, CD40 could enhance the phagocytic activity of macrophages recovered by CD47‐SIRPα blockade, and a fusion protein with high affinity to bind CD40 and CD47 performed better than either CD47 blockade or CD40 agonist alone in a mouse CT26 tumor model.^360^ Although combination therapy can better treat the disease and reduce toxic or side effects, it should also be noted that there may be antagonism or enhanced toxicity between drugs. For example, drugs aimed at blocking macrophage recruitment or depleting macrophages, such as CCL2 or CSF‐1R inhibitors, may be incompatible with drugs that enhance macrophage antitumor activity like CD40 agonists.^361^ In order to design effective combination therapies that fully exploit drug synergies, an insightful understanding of drug targets and resistance mechanisms is required.

To sum up, macrophage‐targeted therapy has been proven to be a promising therapeutic strategy. However, due to the wide distribution of macrophages throughout the organism and the diversity and complexity of regulatory factors, further understanding of the functional and phenotypic heterogeneity of macrophages in specific microenvironment or disease is needed to improve the efficacy of targeted macrophage therapy and reduce off‐targeting. We comprehensively introduce the signal pathways that regulate macrophages and some key regulators in these pathways that can be used as therapeutic targets. The in‐depth understanding of these signal pathways and molecular targets is important for guiding the development of new therapeutic approaches.

## AUTHOR CONTRIBUTIONS

M.L., M.J.W., Y.J.W., and H.F.Z. conceived and drafted the manuscript. G.N.Z. edited and revised the manuscript. Q.L.G. supervised and revised the manuscript. All authors read and approved the final manuscript.

## CONFLICT OF INTEREST STATEMENT

The authors declare no conflicts of interest.

## ETHICS STATEMENT

No ethical approval was required for this study.

## Data Availability

Not applicable.
